# m6aViewer: software for the detection, analysis, and visualization of *N*^6^-methyladenosine peaks from m^6^A-seq/ME-RIP sequencing data

**DOI:** 10.1261/rna.058206.116

**Published:** 2017-10

**Authors:** Agne Antanaviciute, Belinda Baquero-Perez, Christopher M. Watson, Sally M. Harrison, Carolina Lascelles, Laura Crinnion, Alexander F. Markham, David T. Bonthron, Adrian Whitehouse, Ian M. Carr

**Affiliations:** 1 Section of Genetics, Institute of Biomedical and Clinical Sciences, School of Medicine, University of Leeds, St James's University Hospital, Leeds LS9 7TF, United Kingdom; 2 School of Molecular and Cellular Biology, Faculty of Biological Sciences and Astbury Centre of Structural Molecular Biology, University of Leeds, Leeds LS2 9JT, United Kingdom; 3 Yorkshire Regional Genetics Service, St James's University Hospital, Leeds LS9 7TF, United Kingdom

**Keywords:** m^6^A, RNA methylation, peak-calling, m^6^A-seq, next-generation sequencing

## Abstract

Recent methods for transcriptome-wide *N*^6^-methyladenosine (m^6^A) profiling have facilitated investigations into the RNA methylome and established m^6^A as a dynamic modification that has critical regulatory roles in gene expression and may play a role in human disease. However, bioinformatics resources available for the analysis of m^6^A sequencing data are still limited. Here, we describe m6aViewer—a cross-platform application for analysis and visualization of m^6^A peaks from sequencing data. m6aViewer implements a novel m^6^A peak-calling algorithm that identifies high-confidence methylated residues with more precision than previously described approaches. The application enables data analysis through a graphical user interface, and thus, in contrast to other currently available tools, does not require the user to be skilled in computer programming. m6aViewer and test data can be downloaded here: http://dna2.leeds.ac.uk/m6a.

## INTRODUCTION

*N*^6^-methyladenosine (m^6^A) is one of the most abundant internal modifications in polyadenylated mRNAs, but much remains to be learned about its biological roles. Reversible m^6^A methylation may control many aspects of RNA life cycle, affecting transcription, mRNA transport, splicing, stability, transcript abundance, and translation (Meyer and Jaffrey 2014). Moreover, a significant proportion of m^6^A methylation sites are concentrated in the 3′UTR of transcripts, with evidence to suggest that reversible m^6^A methylation regulates miRNA-related pathways (Alarcón et al. 2015; Chen et al. 2015a). Aberrant m^6^A modification patterns have also been linked to diverse human diseases, including infertility, various forms of cancer, alcoholism, obesity, diabetes, and depression, among others (Zheng et al. 2013; Meyer and Jaffrey 2014; Zhao et al. 2014; Du et al. 2015).

**FIGURE 1.**
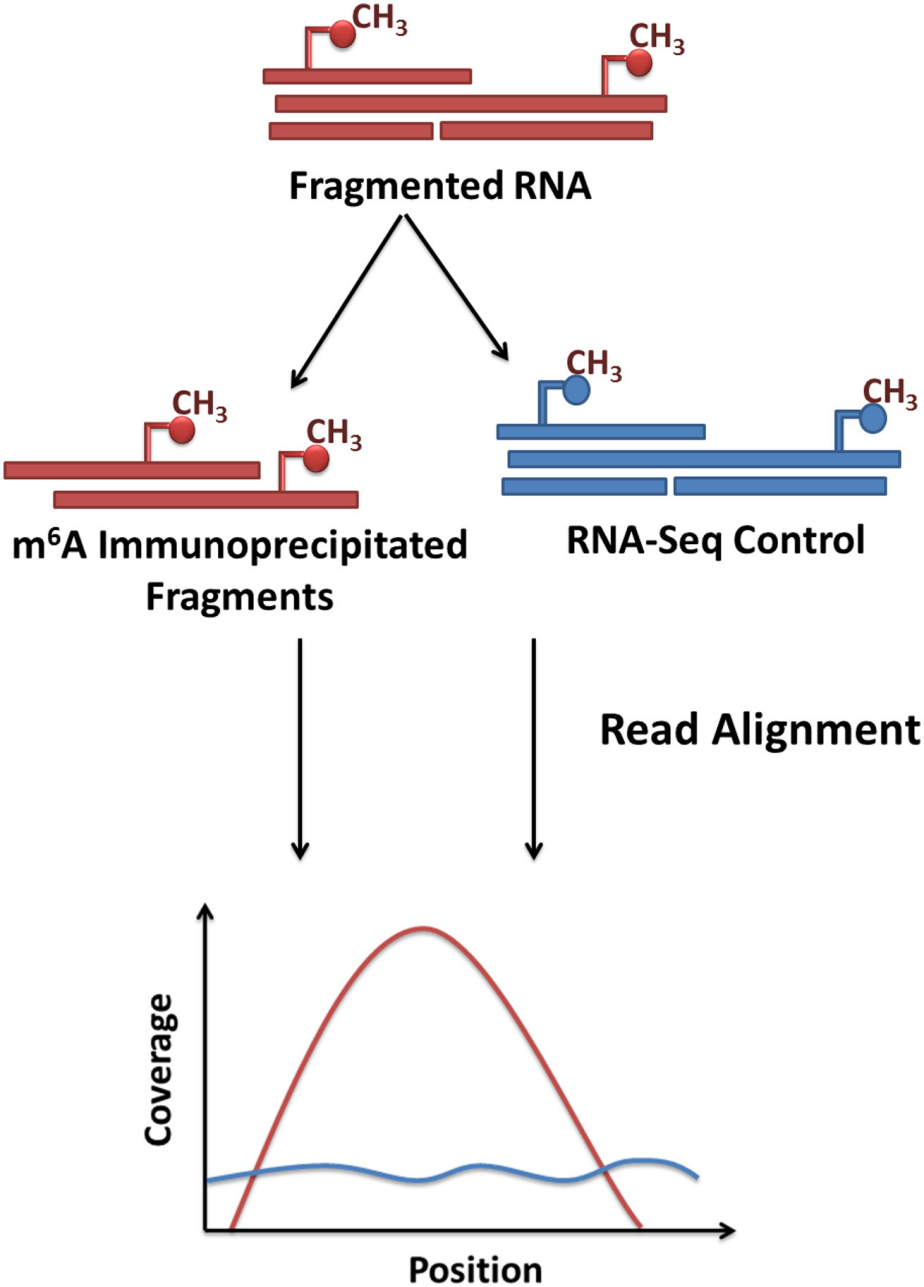
Principle of transcriptome-wide m^6^A profiling using m^6^A-seq. RNA is fragmented and fragments containing methylated adenosines immunoprecipitated using an anti-m^6^A antibody. Fragments are sequenced together with a matched RNA-seq control and all sequenced reads are aligned to the reference transcriptome. Reads from the immunoprecipitated experiment pile up into distinct peaks that show enrichment over RNA-seq control, indicating the presence of a methylated adenosine residue.

In order to explore further biological functions of m^6^A methylation, it is necessary to define how the m^6^A landscape changes in response to varying physiological conditions. Until recently, however, detailed insights have proved elusive due to the lack of scalable methods for transcriptome-wide profiling of m^6^A residues. In 2012, a method based on affinity-capture coupled with “next-generation” sequencing (NGS) was proposed (Dominissini et al. 2012; Meyer et al. 2012). In brief, RNA is fragmented and sequences containing methylated adenosines are recovered using an anti-m^6^A antibody. In addition to coverage biases (such as GC content) seen in DNA sequencing, immunoprecipitated RNA read coverage is also strongly affected by gene expression, and as such, must be compared to a control. Consequently, immunoprecipitated fragments are sequenced together with a matched RNA-seq control and aligned to the reference sequence. The position of a modified residue is indicated by a peak in the coverage distribution of reads from the immunoprecipitated sample when compared to the control data; this peak is expected to be roughly twice as wide at its base as the sequenced fragment length (Fig. 1).

Due to the requirement for a gene expression control as well as the presence of splice-site spanning reads, m^6^A-seq data cannot be readily analyzed by most of the methods used for peak detection in ChIP-seq data, although a protocol adapting the peak-calling software MACS2 (Zhang et al. 2008) does exist. Currently, dedicated software for the analysis of m^6^A data is largely limited to the R environment, with exomePeak package (Meng et al. 2013) and its extensions, HEPeak (Cui et al. 2015) and MeTPeak (Cui et al. 2016), although a number of different in-house approaches to m^6^A peak-calling have been previously described (Dominissini et al. 2012; Meyer et al. 2012; Li et al. 2013). These methods rely on a principle of “binning,” wherein the transcriptome is divided into small, equally sized regions, each of which is tested for the alternative hypothesis that the number of reads in the immunoprecipitated sample is higher than in the control RNA-seq sample. Approaches described by Dominissini et al. (2012), Meyer et al. (2012), and Meng et al. (2014) rely on individual statistical tests and subsequent merging of significantly enriched consecutive bins into a larger region, while HEPeak/MeTPeak (Cui et al. 2015, 2016) models these bins using a hidden Markov model. However, significantly enriched regions identified in this manner can span several kilobases, which make pinpointing the methylated residue(s) a nontrivial task.

In addition, m^6^A-seq data are typically noisy, with a high proportion of false-positive peak calls. For example, in the data shown by Dominissini et al. (2012), in excess of 40,000 unique peaks were identified across three individual replicates; however, only a minority (13,471) of these regions was common to all three replicates. Although the poor reproducibility in this example may also be largely due to the nascency of NGS technologies at the time of the experiment (very short, single-end reads, low coverage), the high level of noise in immunoprecipitated RNA-seq data may arise independently of sequencing platform, for example, from nonspecific antibody binding, DNA contamination, alignment errors due to low complexity sequence regions, or sampling effects due to heteroscedasticity in low expression transcripts.

To address these limitations, we have developed m6aViewer—a Java application for the detection and visualization of m^6^A peaks from sequencing data. m6aViewer provides a flexible m^6^A-seq data analysis and exploration platform via a graphical user interface and aims to facilitate detection of m^6^A sites at close to single-nucleotide resolution. As well as increasing the precision with which modified bases can be identified, m6aViewer also provides methods for reducing the high false-positive rate seen in the typical analysis. The software enables several ways to visualize the m^6^A-seq data (Supplemental Fig. 1), including a peak browser interface.

## RESULTS AND DISCUSSION

### Peak-calling performance

m6aViewer aims to facilitate identification of m^6^A residues at finer resolution than can achieved by currently available software. However, it is difficult to objectively evaluate the performance of any algorithm, since there is no m^6^A-seq testing data set within which the locations of all m^6^A residues are known. Therefore, in order to assess the validity of our approach, we have used multiple distinct test metrics.

The tight colocalization of m^6^A “RRACH” consensus motif with detected peak positions can confer confidence to the peak-calling method, and distance to the nearest m^6^A consensus has been previously used as an m^6^A peak-calling performance metric (Meng et al. 2013). Furthermore, assuming that the consensus sequence motifs are likely to coincide with the actual sites of the methylated residues, the distance to the nearest consensus can illustrate peak-calling precision.

**FIGURE 2.**
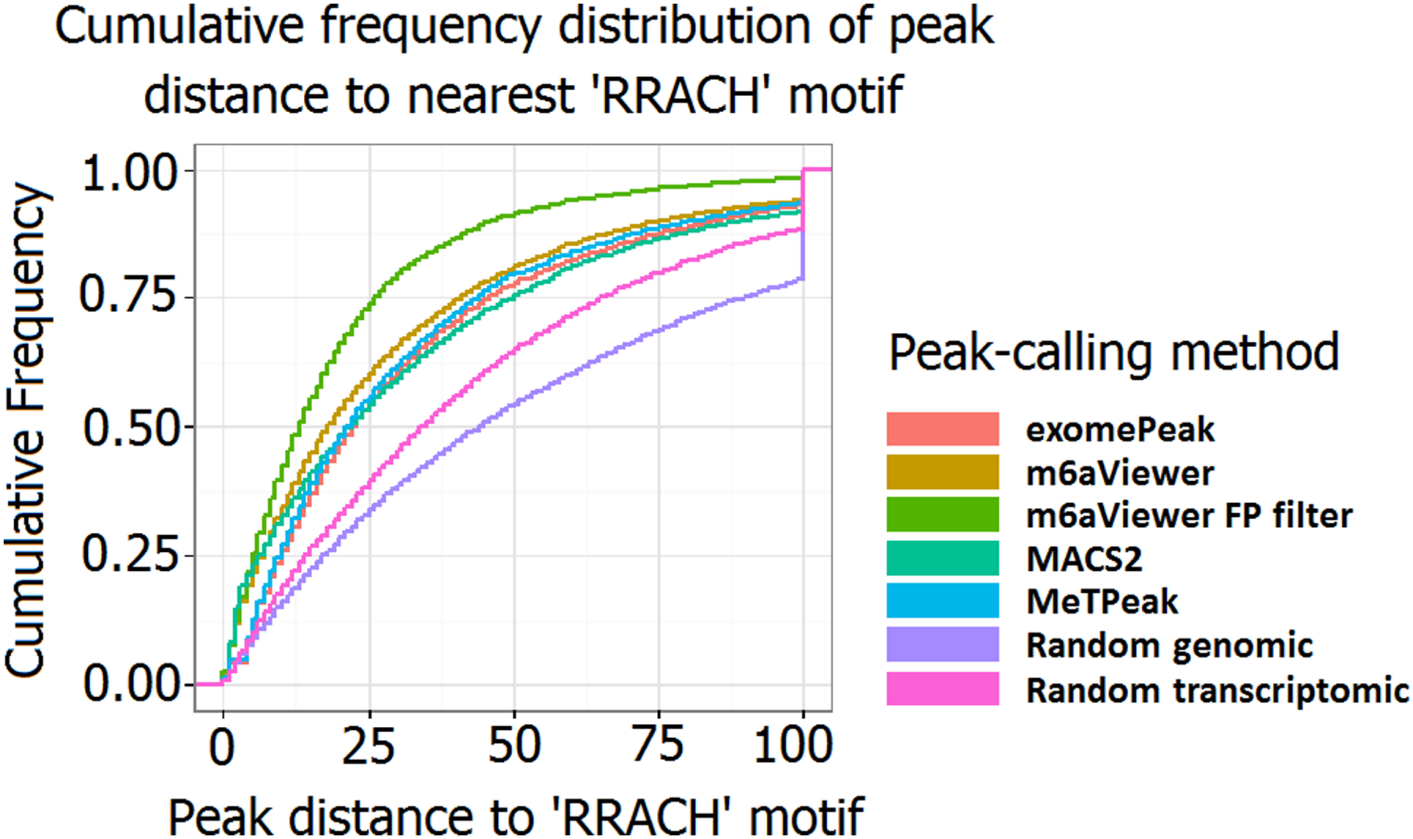
Cumulative frequency distribution of detected peak distance to nearest “RRACH” consensus sequence motif in peaks called by different peak-calling software.

Figure 2 and Supplemental Figure 4 compare the peak to the nearest m^6^A consensus distances between peaks detected by m6aViewer, exomePeak, MeTPeak, MACS2, and randomly selected transcriptomic or genomic control sites. Peaks detected by m6aViewer show high levels of enrichment for previously reported consensus motifs, with known motifs appearing much more frequently near peak positions than near randomly selected transcriptomic positions. The significance of this observation was confirmed by performing a Kolmogorov–Smirnov test for the alternative hypothesis that the cumulative distribution function of m^6^A peak distance to the nearest consensus lies above that obtained from randomly selected transcriptome positions (*P* < 2.2 × 10^−16^, statistic *D* = 0.2474). Peaks detected by all algorithms tested are overall closer to a “RRACH” consensus than the control sites, with MACS2 and m6aViewer called peaks closer to the m^6^A consensus than MeTPeak and exomePeak calls. As MeTPeak and exomePeak output significantly enriched regions, we have used the center of these intervals as the peak position; thus, while these points are not entirely comparable to MACS2 or m6aViewer peaks, it nonetheless serves to demonstrate the difference in peak-calling resolution achieved by these methods.

**FIGURE 3.**
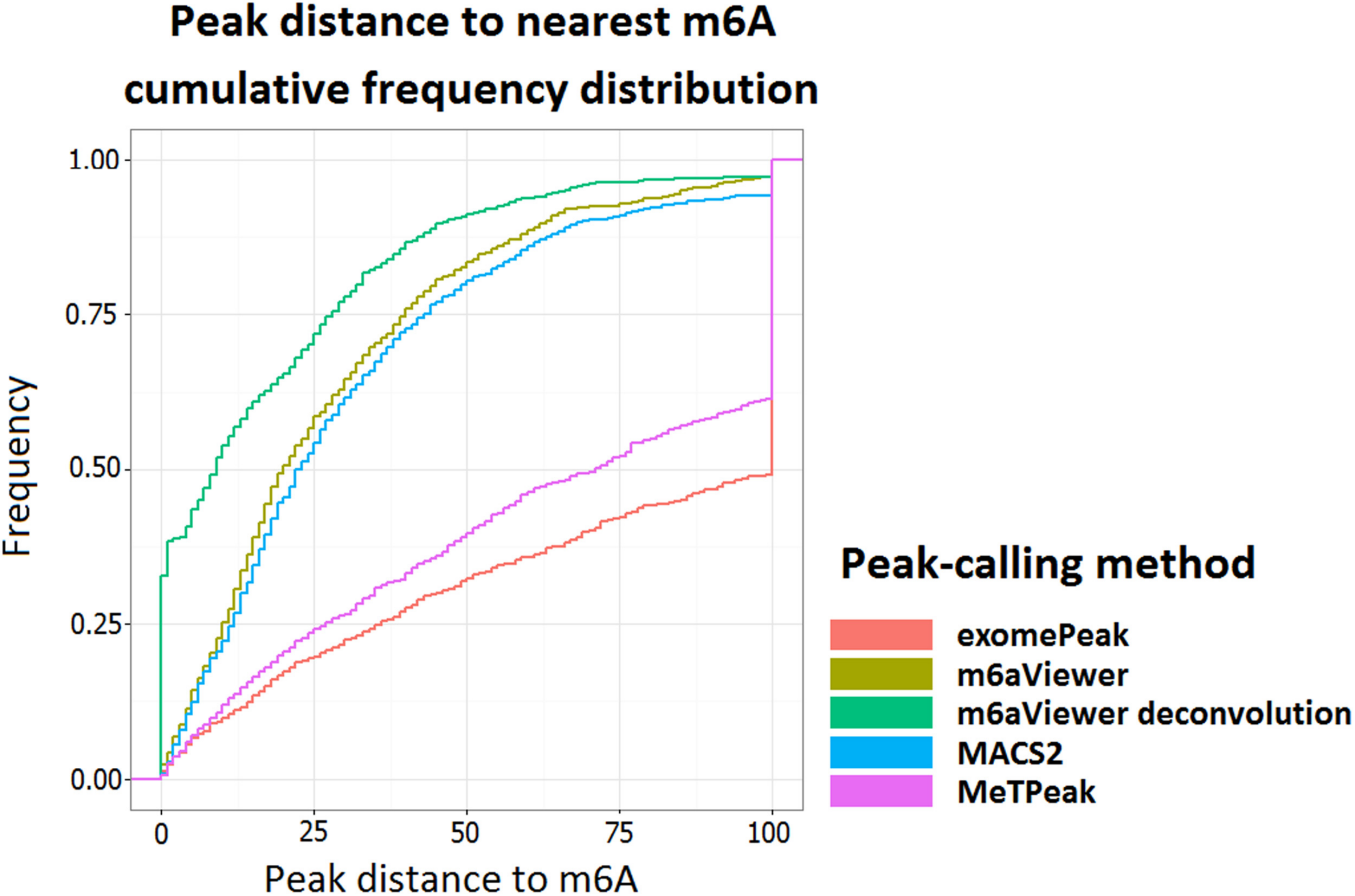
A comparison between m^6^A peak-calling resolutions of multiple m^6^A detection tools. m6aViewer default running mode identifies peaks at a similar precision to MACS2, which is expected as both methods aim to identify peak summits. The center point of regions called by exomePeak and MeTPeak, however, are only rarely indicative of the precise m^6^A position. m6aViewer's peak deconvolution approach results in greatly increased peak-calling resolution.

In addition to exploring consensus motif distributions near detected peaks, we performed a comparison between m6aViewer, exomePeak, MeTPeak, and MACS2 using data from Linder et al. (2015) (Fig. 3). We found that peak positions identified by m6aViewer were overall slightly closer to the methylated adenosine than those identified by MACS2, with 32% of all peaks being called within the distance of 10 nt from the methylated residue by m6aViewer and 25% by MACS2. However, we believe this discrepancy is largely due to the different treatment of paired-end reads and the overall peak-calling precision between MACS2 and m6aViewer is more comparable. That is, the MACS2 version used in our tests could not effectively compute the fragment size of paired reads, because a large proportion of mate-pairs map to different exons several kilobases apart, a feature not usually presented by ChIP-seq data. As expected, center points of regions called by exomePeak and MeTPeak are also overall much further away from m^6^A positions in this data set. Furthermore, it is worth noting that any inaccuracies present in our testing data set may be reflected in the results. The residues reported by Linder et al. (2015) are likely to contain some false positives. Conversely, it is feasible that not all clustered m^6^A residues are present in the correspondingly enriched m^6^A-seq regions. However, currently no matched single-nucleotide resolution m^6^A map and m^6^A-seq data sets are available.

We tried to use comparable peak-calling parameters with exomePeak, MeTPeak, MACS2, and m6aViewer, but there is considerable scope for modification of the analysis. As a result of these uncertainties, we consider it beyond the scope of this work to attempt a more extensive comparison between the different methods.

### Peak deconvolution

Our results highlight that the called m^6^A-seq peak summits very rarely precisely correspond to the actual site of methylation, a pattern also noted by Linder et al. (2015). This difficulty can be further compounded by the presence of multiple methylated sites in close proximity, which blurs the expected peak signal. Here, we show that it is possible to improve the precision with which m^6^A residues are called by modeling each region as a mixture of fragment coverage distributions. Our peak deconvolution approach correctly identified the position of a methylated residue in 34% of cases tested, compared to 1%–3% by methods (including default m6aViewer setting) considering peak summits alone (Fig. 3). Furthermore, it is possible to identify multiple methylated residues present in close proximity. For instance, Supplemental Figure 2 shows two methylation sites reported by Linder et al. (2015) giving rise to a single broad peak; using our probabilistic model to cluster individual RNA fragments, m6aViewer can correctly identify these sites as the most likely to fit the observed data.

### False-positive peak filter

As it has been demonstrated that false-positive peaks can constitute a large proportion of all peaks detected in an m^6^A-seq experiment, we have sought to see if we could identify and filter out these events without compromising the recall of genuine m^6^A sites.

Recently, a number of machine learning models for computational identification of m^6^A sites from sequence features were proposed. Zhou et al. (2016) implemented SRAMP, a web server for m^6^A site prediction from transcript sequence in a number of mammalian cell types. SRAMP combines multiple random forest predictors trained on sequence features from known m^6^A sites. A similar web tool implementing a support vector machine predictor has been developed for yeast data (Chen et al. 2015b).

**FIGURE 4.**
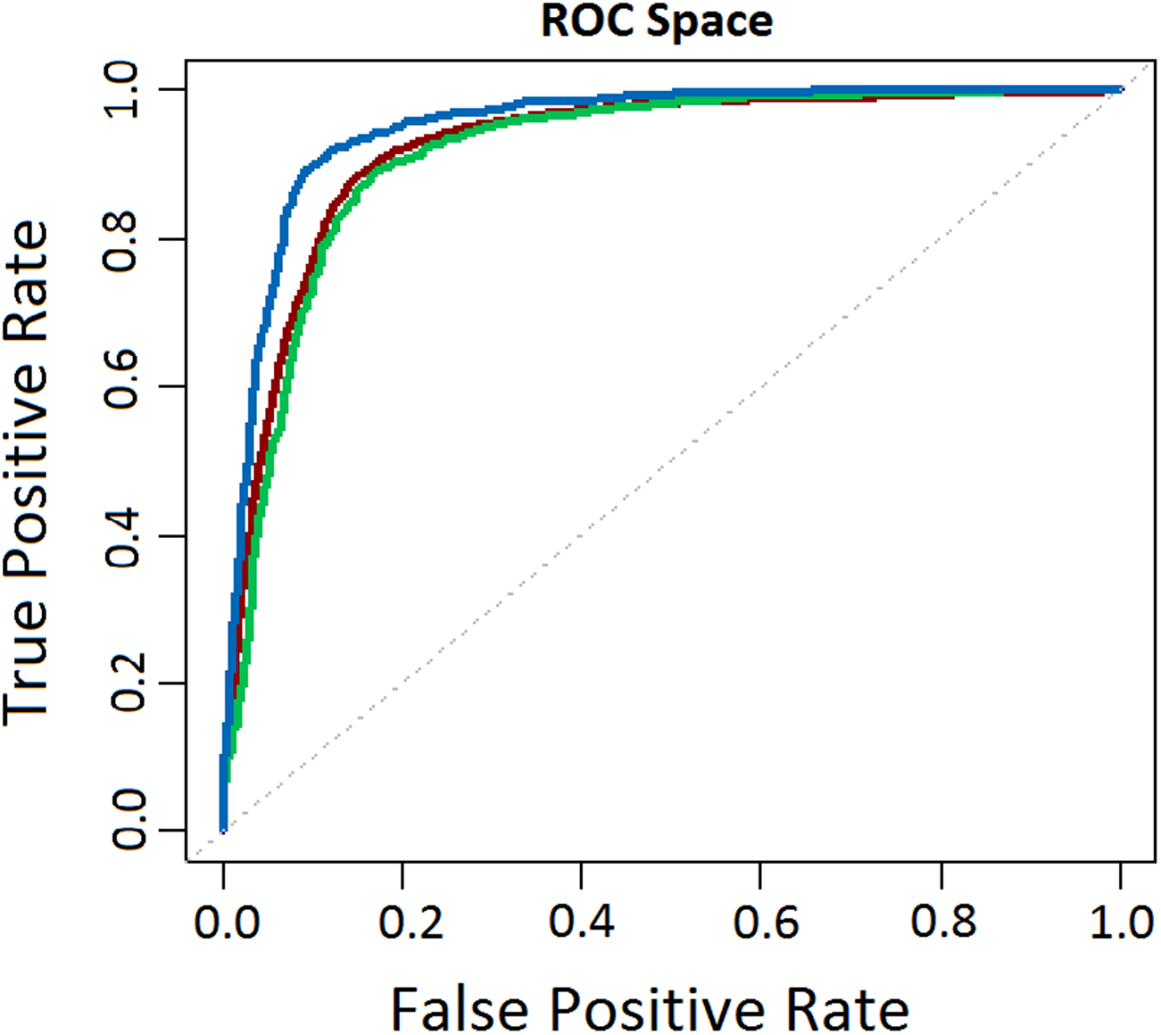
ROC analysis showing the performance of m6aViewer's false-positive filter classifier when assessed using 10-fold cross-validation of the training data set (AUC = 0.923, dark red), independent A459 cell line testing data set (AUC = 0.95, blue), and independent mouse fibroblast testing data set (AUC = 0.913, green).

Here, we implement a supervised learning filter that combines an MTD RNA sequence model with a feature-based Random Forest learner. Our combined model can distinguish genuine m^6^A sites with high accuracy, with 10-fold cross-validation of our model achieving area under the receiver operating characteristic (ROC) curve (AUC) of up to 0.923. We show that our approach can be applied to different tissue types (AUC = 0.95, independent A459 cell test set), as well as different mammalian species (AUC = 0.913, independent mouse fibroblast test set) (Fig. 4). However, similarly to the work by Zhou et al. (2016), our approach is also unlikely to generalize well to more distantly related species.

In contrast to the methods proposed by Zhou et al. (2016) and Chen et al. (2015b), we use false-positive peaks as our negative training examples, rather than randomly selected transcriptomic positions. Despite our best efforts to obtain a high-confidence training data set, it is likely that some inaccuracies remain. While the training examples were obtained from cell-type matched m^6^A-seq data sets to minimize the effects of biological variation, it is likely that a proportion of training instances is in fact mislabeled. Additionally, while generally robust, methyltransferase knockdown does not abolish the presence of m^6^A methylation completely; this is likely to also contribute to mislabeled training instances. High classification accuracy, however, suggests that our approach is resistant to noise in the training data, with the positive and negative instances overall forming sufficiently biologically distinct groups.

**FIGURE 5.**
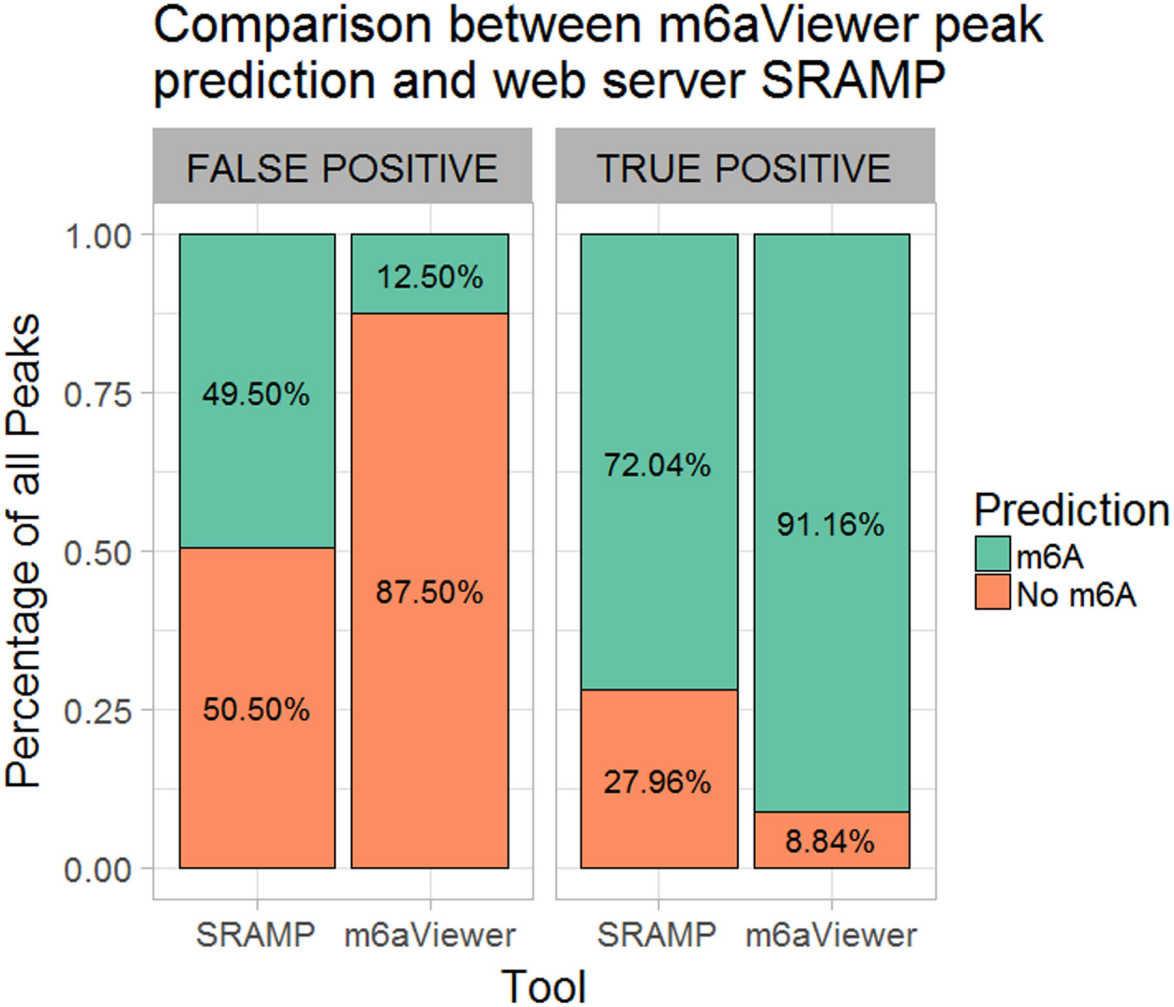
Comparison between m^6^A predictions made by web server SRAMP and m6aViewer. SRAMP predicted m^6^A within peak sequences from the true positive subset in 72.04% of cases, while m6aViewer classified these as true positives in 91.16% of cases. In the false-positive peak subset, SRAMP predicted m^6^A residues in 49.5% of cases, while m6aViewer classified 12.5% of these as true positives.

To assess how well m6aViewer's false-positive filter compares with sequence-based predictions of SRAMP, we have used the SRAMP web server to predict m^6^A positions within peak sequences from our A459 cell line testing data set. SRAMP predicted m^6^A residues to be present in 72.14% of all instances labeled as true positive m^6^A peak sequences, while m6aViewer classified 91.14% of these sequences as such (Fig. 5). In the false-positive peak subset, SRAMP predicted m^6^A to be present in nearly half the sequences, while m6aViewer misclassified these in 12.5% of total instances. As discussed previously, this discrepancy could be partially explained by inaccuracies in our data set, where genuine m^6^A sites could be mislabeled as false positives. Furthermore, SRAMP was run with default settings—a running mode that is faster, but does not consider predicted secondary RNA structure information, which reportedly enhances classification performance. These results, however, are not surprising, as SRAMP effectively detects the potential for m^6^A methylation in a given sequence, whereas m6aViewer aims to frame these predictions in an experimental context by considering m^6^A-seq data features. As such, some adenosines encompassed by our 200-nt false-positive peak sequences could be potentially methylated under certain conditions due to the dynamic nature of m^6^A; thus, SRAMP's predictions would be correct within the designed scope of the software. Indeed, this potential for methylation may be a major contributing factor for the much lower discriminatory power of our sequence-only model.

**FIGURE 6.**
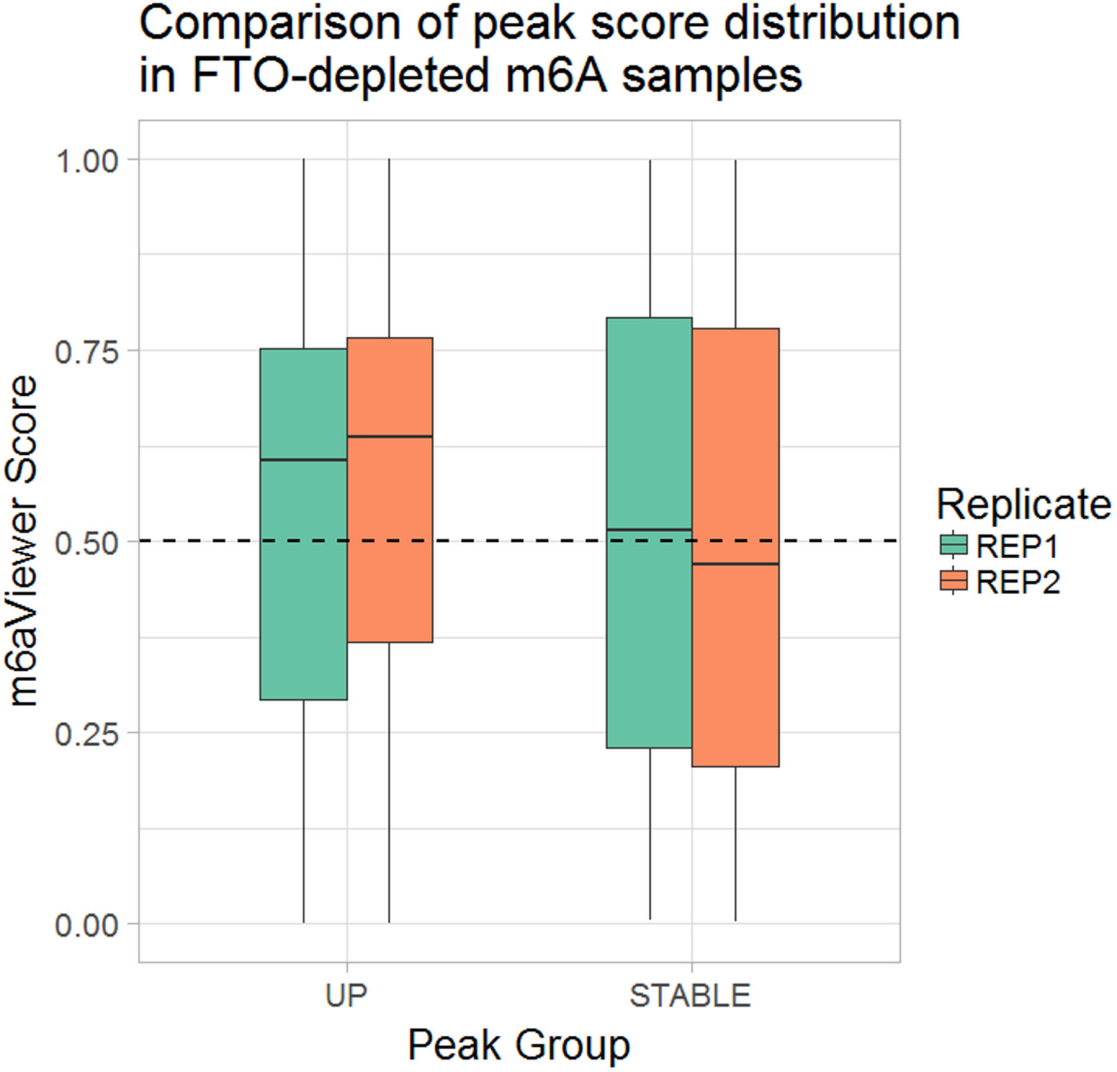
m6aViewer's false-positive filter classifier score distribution in peaks from two FTO-depleted and matched control sample replicates. Two subsets of peaks are compared, a “STABLE” (peaks that show no change in enrichment between FTO-depleted and control samples) subset and an “UP” (peaks that show at least a twofold increase in peak enrichment in FTO-depleted samples over control samples) subset. In replicate 1, 51.21% of stable peaks and 65.64% of up-regulated peaks were scored 0.5 or higher; in replicate 2, the difference between score distributions was more pronounced, with 47.87% of stable and 70.13% of up-regulated peaks scoring 0.5 or higher. At a lower threshold of 0.4, in replicate 1, 71.26% of up-regulated peaks and 58.78% of stable peaks are classified as true positives; in replicate 2, 54.28% of stable and 75.34% of up-regulated peaks are classified as true positives.

In order to further ascertain whether m6aViewer's false-positive filter provides biologically meaningful results, we have performed peak-calling in an RNA demethylase FTO-depleted and matched control m^6^A-seq data sets. From the detected peaks, we selected those that could be considered stable (detected in both FTO-depleted and control samples and change in enrichment between FTO and KO is less than 0.5-fold) or more enriched in the FTO-depleted sample (detected in both FTO-depleted and control samples and IP enrichment over INPUT at least twofold higher in the FTO-depleted sample). These two groups of peaks were then classified into false positives and true positives using m6aViewer, and a classifier score was assigned to each peak (0–0.5 indicating likely false positives, 0.5–1.0 indicating true positives, with increasing confidence as score approaches 1.0). We observed that peaks that show an increase in enrichment in FTO-depleted samples are more likely to be scored as true positives by our classifier (Fig. 6). This observation is in line with the hypothesis that genuine m^6^A peaks are more likely to respond to RNA demethylase depletion treatment, whereas peaks that appear in the data due to experimental artifacts are less likely to be thus affected.

The final classifier model is implemented as an optional peak filter and allows the user to bias the false-positive filter toward precision or recall. Our cost/benefit analysis (Supplemental Fig. 3) suggests that using default settings, m6aViewer can filter out 86.02% of false-positive peaks at the cost of losing 9.23% genuine m^6^A sites.

### Implementation and availability

m6aViewer is made available through a cross-platform graphical user interface requiring Java 1.8 (or greater) to run and thus requires no command-line or programming knowledge to use. The basic peak-calling functions require only indexed and sorted BAM files as input; however, the user may provide a GTF annotation file and reference sequences in fasta format to take advantage of additional analysis and annotation options (for users unfamiliar with command line interface, a number of services, such as Galaxy [Goecks et al. 2010], exist that facilitate read alignment, sorting and indexing steps through a web-based interface). Moreover, unlike exomePeak, our peak-calling method is uncoupled from genomic annotations, and as such, can be applied to sequencing data from organisms with poorly annotated genomes, or for peak detection in novel transcripts.m6aViewer allows the user to comparatively visualize peaks across multiple samples (Supplemental Fig. 1A). The peak browser interface provides convenient, interactive visualization of the peaks in the context of genome annotations and nearest consensus motif sequences. The methylation landscape can be viewed at chromosome level using an ideogram view (Supplemental Fig. 1B), which provides local mouse zoom. Custom chromosomes can be drawn by providing a cytoband data file in a tabular format (available from UCSC). Additionally, multiple data summary statistics can be plotted by m6aViewer, including peak distribution and sample-to-sample peak overlap heat charts.

Peak-calling results can be easily exported to a tab-delimited output text file. m6aViewer outputs one text file per sample containing positions, significance and enrichment values of all detected peaks. Additionally, the file may also contain gene annotation, nearest consensus motif annotation or false-positive filter confidence score, depending on the peak-calling settings used. In the case of multiple samples, an additional file is output that contains pairwise peak comparisons between all samples. Each peak position is matched across samples and peak data are collated to provide a convenient output format for further analysis. For each pairwise sample, log fold enrichment change is also computed to aid quick identification of peaks exhibiting the biggest changes in methylation.

m6aViewer is highly customizable; however, sensible default parameters are in place that can be readily used for most analyses. Extended details about software usage are provided in online application documentation. m6aViewer Java application and test data can be downloaded at http://dna2.leeds.ac.uk/m6a.

## MATERIALS AND METHODS

### Processing of sequence data

m6aViewer requires sorted and indexed BAM files as input; as such, raw sequence data preprocessing and alignment steps are important. Here, all sequence data were preprocessed using Cutadapt software (Martin 2011) in order to remove sequencing adapter contamination and poor quality bases. Sequencing reads were then aligned to the human hg19 or mouse mm10 reference genomes. Reference gene annotation was downloaded from the UCSC Genome Browser database using the table browser tool, while reference sequences were downloaded from the UCSC ftp site (Kent et al. 2002; Karolchik et al. 2004). Processed reads were aligned using STAR (Dobin et al. 2013), a splicing-aware aligner. The resulting alignments were sorted and indexed using SAMtools (Li et al. 2009) in order to obtain BAM files for downstream peak-calling.

Precise determination of sequenced RNA fragment coverage is essential for m^6^A peak-calling; however, typical sequencing reads do not always accurately represent the sequenced molecule. Single-end reads are often shorter than the length of the immunoprecipitated fragment, and thus can result in a shifted (stranded library preparation protocol) or bimodal (nonstranded library preparation) coverage distribution of reads. Similarly, paired-end reads may not cover the center of the fragment, or may overlap, resulting in artifactual inflation of coverage at certain positions. m6aViewer counts fragment (rather than read) coverage distribution to account for these biases. Since paired-end sequencing sequences each RNA fragment from both ends, fragment length and position information can be easily inferred. However, single-end reads are extended in the direction of the 3′ end of the read up to a user-specified fragment size.m6aViewer also takes into account split reads when calculating fragment coverage distribution, and any read extensions will respect intron–exon boundaries, provided a gene transfer format (GTF) annotation file is imported. Improper treatment of paired-end read mapping across multiple exons can be particularly problematic when using ChIP-seq peak-calling software to call m^6^A peaks, since gapped alignments are not the norm in DNA sequencing.

Sequenced reads can be optionally filtered to ensure that only the highest quality alignments are used for subsequent peak-calling. This includes skipping reads flagged as PCR/optical duplicates, alignments below a certain mapping quality threshold, secondary alignments, and in the case of paired-end sequencing, improper read pairs. For further details on sequence data processing, see Supplemental Methods.

### m^6^A peak-calling

Base-level RNA fragment coverage counts are extracted from immunoprecipitated (IP) and matched control (INPUT) sample BAM files, and coverage between IP and INPUT is scaled to library size. The signal is smoothed using a sliding window approach and initial candidate peak positions are identified by finding all the local maxima in the IP coverage distribution. The local maxima are detected as a change in the coverage distribution gradient over the expected peak width, where a position (or a run of positions at equal coverage) is considered a local maximum if preceding base coverage follows an increasing trend and successive base coverage is decreasing. The expected peak width is estimated as twice the median sequenced fragment length in the case of paired-end reads, or from a user supplied fragment size (default: 100 nt) in the case of single-end reads.

Multiple m^6^A residues can exist in close proximity to one another (Linder et al. 2015), resulting in fully or partially overlapping peaks (Supplemental Fig. 1A). m6aViewer can identify these events by identifying multiple local maxima in close proximity separated by a local minimum in coverage distribution. While m^6^A residues that produce a signal resulting in completely overlapping peaks cannot be resolved by this approach, peaks that only partially overlap can be identified, and are treated separately. This is in contrast to binning-based approaches used by software such as exomePeak (Meng et al. 2013), in which a single significant region can encompass multiple distinct peaks.

As the number of replicates for m^6^A-seq experiments is typically low, Fisher's exact test is used to determine significantly enriched peaks for each replicate individually. Each local maximum identified is tested against the null hypothesis that the read distribution in the immunoprecipitated sample is not higher than that in the control using total fragment counts aligning to the peak region. The peak region is defined as the region encompassing the number of bases equal to the sequenced fragment/insert length to each side of the detected maximum; in cases of peak overlap, the region boundary to the overlapping side(s) of the peak is defined as a midpoint between the two peaks. This allows the analysis to take into account the total number of reads in IP and INPUT samples, providing a statistical confidence level for each peak. To correct for the large number of statistical tests carried out, m6aViewer implements several alternative ways of controlling false discovery rate (FDR). For further details, see Supplemental Methods.

In order to facilitate analyses that focus on detecting highest confidence m^6^A peaks, in addition to *P*-value and FDR-based cutoffs, m6aViewer also implements IP enrichment (default: twofold) and coverage (default: 20 reads) filters to increase the specificity of peak-calling. The former allows the elimination of peaks that show only low levels of enrichment, while the latter filters out peaks that are called from a low number of reads spanning the region (but that are still statistically significant). The default values favor retaining high-confidence peaks only, but can be freely adjusted using the options menu. Additionally, an optional (default: off) reproducibility filter can be applied in cases where multiple sample replicates are available. This compares all detected peaks within a replicate group, retaining only those that are reproducible. This option requires samples to be grouped via the GUI menu prior to peak-calling.

### Peak deconvolution

Peaks arising from multiple m^6^A sites in very close proximity can often be visually indistinguishable from single sites, and thus such cases cannot be identified using the approach described in the “m^6^A peak-calling” section. Conversely, the summit of detected single m^6^A peaks rarely precisely corresponds to the site of methylation. Here, we propose a mixture distribution-based approach to deconvolute overlapping peaks and pinpoint m^6^A methylation sites with increased precision. The method is implemented as an alternative (slower) running mode for m6aViewer.

Briefly, the observed fragment coverage distribution in an enriched region can be thought of as a mixture of coverage distributions, each arising either from antibody binding to a methylated residue or confounding “noise” reads that may arise from sources such as sequencing/alignment errors or RNA molecules randomly “sticking” to beads and other surfaces during NGS library preparation. Thus, for each such region, we try to find and fit a mixture of *n* fragment coverage distributions that best explain the observed data. We use an expectation maximization (EM) approach to iteratively compute the likeliest m^6^A sites. During EM, we iteratively switch between computing the probability distribution of RNA fragments “belonging” to each putative m^6^A position and reestimating said m^6^A positions and corresponding mixture proportions, until convergence. For further details on the expectation maximization algorithm, see Do and Batzoglou (2008).

Initializing the EM algorithm to appropriate values is crucial to its performance, and a number of initializing techniques have been proposed (Maitra 2009; Melnykov and Melnykov 2012). Given that all possible methylation sites can be obtained directly from the reference sequence—that is, methylation in this context can only occur at an adenosine residue (and is more likely to occur within a consensus sequence)—we iteratively select a number of these positions which account for the most mapped fragments in the region, akin to the method described in Melnykov and Melnykov (2012). Similarly, mixture probabilities can be initialized as a normalized proportion of fragments mapped to each initial position.

It is particularly important to estimate correctly how many peaks should be fitted. While fitting a larger number of peaks will always result in an increase in log-likelihood for the model, this will often result in over-fitting. We use Bayesian information criterion (BIC) to select the model with the likeliest number of peaks in order to account for both the likelihood and the complexity of the model (Schwarz 1978). For further details on m6aViewer's peak deconvolution approach, see Supplemental Methods.

### Identification of false-positive peaks

False-positive peaks can arise due to nonspecific antibody binding, alignment errors, or even RNA/DNA contamination. These peaks can constitute a large proportion of all detected peaks and are problematic in the analysis of m^6^A-seq experiments. Here, we implemented a supervised, ensemble learning filter to aid the distinguishing of true-positive m^6^A sites from false-positive peaks.

We downloaded human methyltransferase knockdown and matched control m^6^A-seq data from the ArrayExpress archive (original experiments: Schwartz et al. [2014], accession: E-GEOD-55572) in order to obtain a set of true-positive and false-positive peaks for use as training data. We used data from HEK293T cells to train and assess our initial model using 10-fold cross-validation, while data from human A549 cells and mouse fibroblast cells were used as independent testing data sets to assess how well our model generalizes to different tissue types. For further validation of our model, we additionally obtained m^6^A-seq data from RNA demethylase FTO-depleted mouse 3T3-L1 cell lines.

In order to obtain a high quality training data set, peaks that could be identified with high confidence as either m^6^A or nonspecific antibody peaks were selected. A peak was considered as a high-confidence m^6^A peak if it was not detected in the knockdown sample. Similarly, a peak was considered to be a technical false positive only if the change in the levels of enrichment between the knockdown and the control was <0.5-fold. The final HEK293T data set consisted of 2098 peaks, 1030 of which are false-positive training instances and 1068 true-positive instances.

In order to see whether true positives could be distinguished from false positives using RNA sequence information alone, we implemented a mixture transition distribution (MTD) model (Raftery 1985) using RNA sequence surrounding each peak as training data. Markov chains are well suited for representing RNA sequences and have been applied to a wide range of DNA and RNA sequence classification problems (Salzberg et al. 1998, 1999; Eddy 2004). Higher order models often produce better results (Narlikar et al. 2013), but are computationally difficult to model because the number of required parameters grows exponentially. MTD allows approximation of high-order Markov chains in linear space by considering the effect of each lag on the conditional probability of observing each base in a sequence separately. Here, we represented peaks as 200-nt sequences of A, T, G, C, and M (where M is the detected methylated adenosine residue) and estimated transition probability matrices for false-positive and true-positive models from a set of training sequences, comprising two-thirds of the entire data set. Model parameters were optimized using the maximization of the log-likelihood approach described by Berchtold and Raftery (2002). This approach enables us to capture the sequential nature of the data, an important aspect that can be lost in feature-based classification. For further details on MTD, see Berchtold and Raftery (2002) and Raftery (1985).

As our sequence-based model alone did not achieve practicable precision and recall rates (AUC = 0.79), we opted to incorporate additional data by combining our sequence-based model with a feature-based approach. We selected 61 features, including secondary RNA structure (Lorenz et al. 2011), position and conservation status of the nearest consensus sites (Karolchik et al. 2004; Siepel et al. 2005), information about putative miRNA binding sites (Kozomara and Griffiths-Jones 2014), transcript-level information such as exon length and whether the peak is within a coding or regulatory region, *k*mer content and peak attributes (e.g., enrichment over INPUT control) and used this information as a basis for a feature-based model. We trained a Random Forest classifier—an ensembl of random decision trees wherein the final classification is decided by the majority vote—using an established Java machine learning library Weka (Hall et al. 2009). The final model was built using 1000 random trees, each considering six random features at each node. For further details on Random Forest algorithm, see Breiman (2001). We found that including predicted RNA secondary structure information in our model—a feature used by Zhou et al. (2016)—did not result in increased performance, thus these features were excluded from the final model. This suggests that either the error rate in RNA secondary structure prediction is too high, RNA secondary structure is not important for RNA adenosine methylation, or a similar RNA secondary structure is present at nonspecific antibody binding sites to that of actual m^6^A sites, which could also be an antibody site recognition factor. For further details on m6aViewer's sequence and random forest models, see Supplemental Methods.

Finally, we have opted to compare m6aViewer's false-positive classifier filter with m^6^A site predictions made by webserver SRAMP (Zhou et al. 2016). As the SRAMP web interface does not allow batch queries, we have automated SRAMP predictions using a custom program to construct HTTP POST queries at SRAMP default settings (no RNA secondary structure information, use all tissues).

### Performance assessment and comparison with other tools

In order to validate our peak-calling approach, we have used m6aViewer to detect peaks in multiple published m^6^A-seq data sets. We compared the results with two other m^6^A peak-calling algorithms, exomePeak (Meng et al. 2013) and its recently described extension MeTPeak (Cui et al. 2016), as well as the ChIP-seq calling tool MACS2 (Zhang et al. 2008), using multiple distinct metrics.exomePeak analysis was run in R using default parameters, while m6aViewer, MACS2, and MeTPeak parameters were set to mirror these defaults where possible (*P*-value: <0.05, minimum enrichment: 1.0, MACS2 command line option “—call-summits”). m6aViewer was run without applying the false-positive filter, unless specifically indicated. Benchmarks were performed using m6aViewer's default running mode, except in cases where peak deconvolution is indicated.

For exomePeak and MeTPeak results, the center point of the reported enriched regions was computed and used as the peak position for all assessments. In contrast, ChIP-seq peak-calling software MACS2 (Zhang et al. 2008) outputs a single peak position corresponding to the detected peak summit. We investigated how m^6^A peak-calling by these algorithms compares to m6aViewer.

As no m^6^A-seq data set exists wherein all m^6^A positions are known, we use peak distance to nearest m^6^A consensus (RRACH) as one measure of algorithm performance. As an expected background control, consensus distance to randomly selected (via random number generator) genomic or transcriptomic positions was computed. In order to increase the robustness of this evaluation, the transcriptomic control positions were selected from peak-matched transcript regions (either CDS, 5′UTR, 3′UTR, or intronic), as randomly selected transcripts may not accurately capture the expected rate of m^6^A consensus occurrence. We applied this measure to an m^6^A-seq data set from HepG2 cells (Dominissini et al. 2012).

Recently, Linder et al. (2015) described a mutation-based technique to obtain a single-nucleotide resolution map of m^6^A methylation sites in HEK293T cells. Here, we use this data to benchmark m^6^A peak-calling resolution. As no matched m^6^A-seq data set is available, we opted to use the HEK293T m^6^A-seq data set from Schwartz et al. (2014). We intersected the HEK293T m^6^A-seq data set with the m^6^A positions reported by Linder et al. (2015) and selected the 1000 highest confidence residues identified in the mutation map that also corresponded to an enriched region in the m^6^A-seq data set. We performed peak-calling on the m^6^A-seq data set using m6aViewer, MACS2, exomePeak, and MeTPeak and compared the precision with which peaks were called.

### Me-RIP data sets

Me-RIP data sets used for the development and performance assessments of m6aViewer were downloaded from the ArrayExpress (Kolesnikov et al. 2015) database. These are as follows: RNA methyltransferase depleted and matched control 50-nt paired-end sequence data from HEK293T, A459 and mouse fibroblast cells (accessions: E-GEOD-55575 and E-GEOD-55572); 36-nt single-end read HepG2 cell line data (accession: E-GEOD-37005); FTO-depleted mouse 3T3-L1 cell line data (accession: E-GEOD-53244); 50-nt single-end HIV-infected and control T-cell data (accession: E-GEOD-74016); and 50-nt H1299 cell line data (accession: E-GEOD-76367). All data were preprocessed as detailed in the “Processing of sequence data” section.

## SUPPLEMENTAL MATERIAL

Supplemental material is available for this article.

## Supplementary Material

Supplemental Material
