# Supplementary material for: m6aViewer: software for the detection, analysis, and visualization of N6-methyladenosine peaks from m6A-seq/ME-RIP sequencing data
Source: RNA. 2017 Oct;23(10):1493–501. doi: 10.1261/rna.058206.116 (PMC5602108; doi:10.1261/rna.058206.116)
Supplement: Supplemental Material [file supp_23_10_1493__index.html]

m6aViewer: software for the detection, analysis, and visualization of N6-methyladenosine peaks from m6A-seq/ME-RIP sequencing data — Supplemental Material 

# m6aViewer: software for the detection, analysis, and visualization of *N*6-methyladenosine peaks from m6A-seq/ME-RIP sequencing data

## Supplemental Material

- Supplemental\_Data.xlsx
- Supplemental\_Figures.pdf
- Supplemental\_Material.pdf
