# Supplementary material for: m6aViewer: software for the detection, analysis, and visualization of N6-methyladenosine peaks from m6A-seq/ME-RIP sequencing data
Source: RNA. 2017 Oct;23(10):1493–501. doi: 10.1261/rna.058206.116 (PMC5602108; doi:10.1261/rna.058206.116)

Supplementary Figure 1A.

m6aViewer’s peak browser interface enables a “comparison view” of peaks across multiple samples. m6aViewer can detect partially overlapping peaks. Tight co-localization of consensus sequence motifs (dotted vertical lines) is shown. Information about each peak, such p-value, is provided via tooltips.

A) m6aViewer's peak browser interface

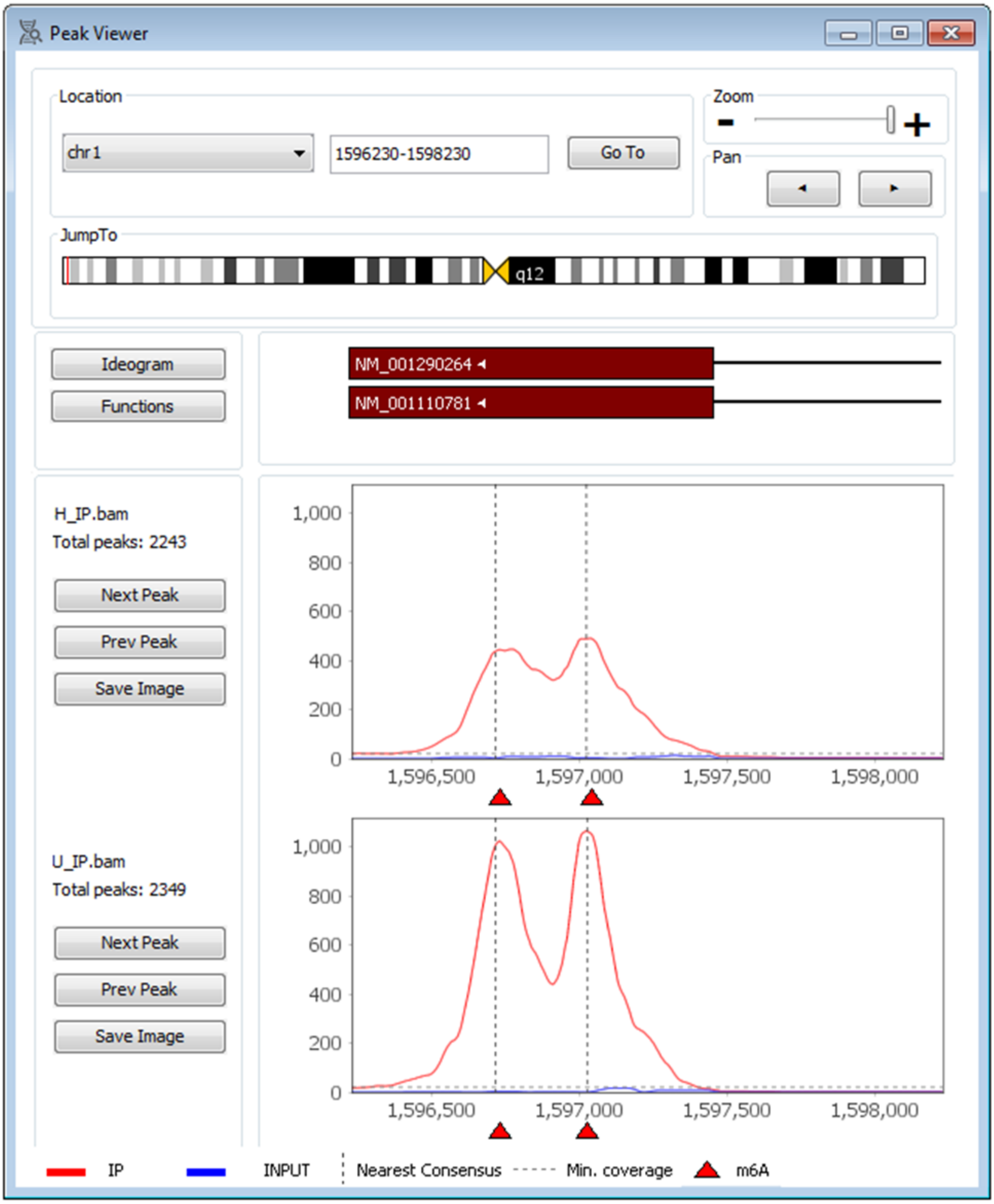

Supplementary Figure 1A.

Peaks can be visualized at chromosome level using m6aViewer's "ideogram view". Here, detected peaks across two samples in human chromosome 22 are shown.

B) m6aViewer's peak ideogram view

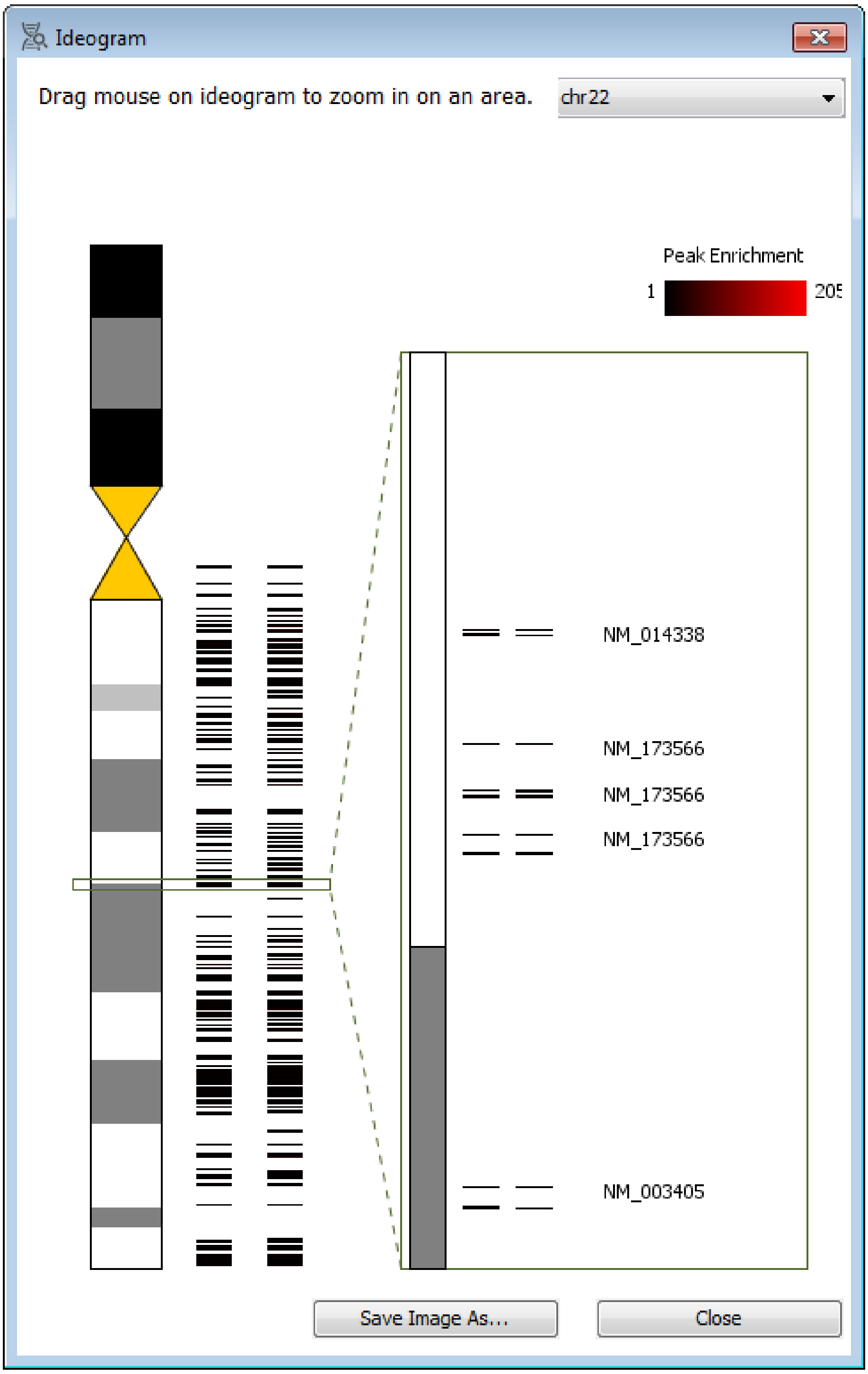

Supplementary Figure 2

An example of m^{6}A peak deconvolution. Linder *et al*. (2015) reported two m^{6}A modifications in SEPN1 gene at chromosome 1 positions 26142428 and 26142500. This region also shows enrichment over input in our test m^{6}A-Seq dataset. After performing peak de-convolution for this region, m6aViewer also identifies positions 26142428 and 26142500 as the most likely methylated sites to give rise to the observed fragment coverage distribution. The observed fragment coverage distribution is shown in yellow; fragments which could not be ascribed to either peak are considered noise and are shown in red; most likely fragment coverage distributions for m^{6}A at positions 26142428 and 26142500 are shown in blue and green respectively. m^{6}A positions are indicated by vertical lines.

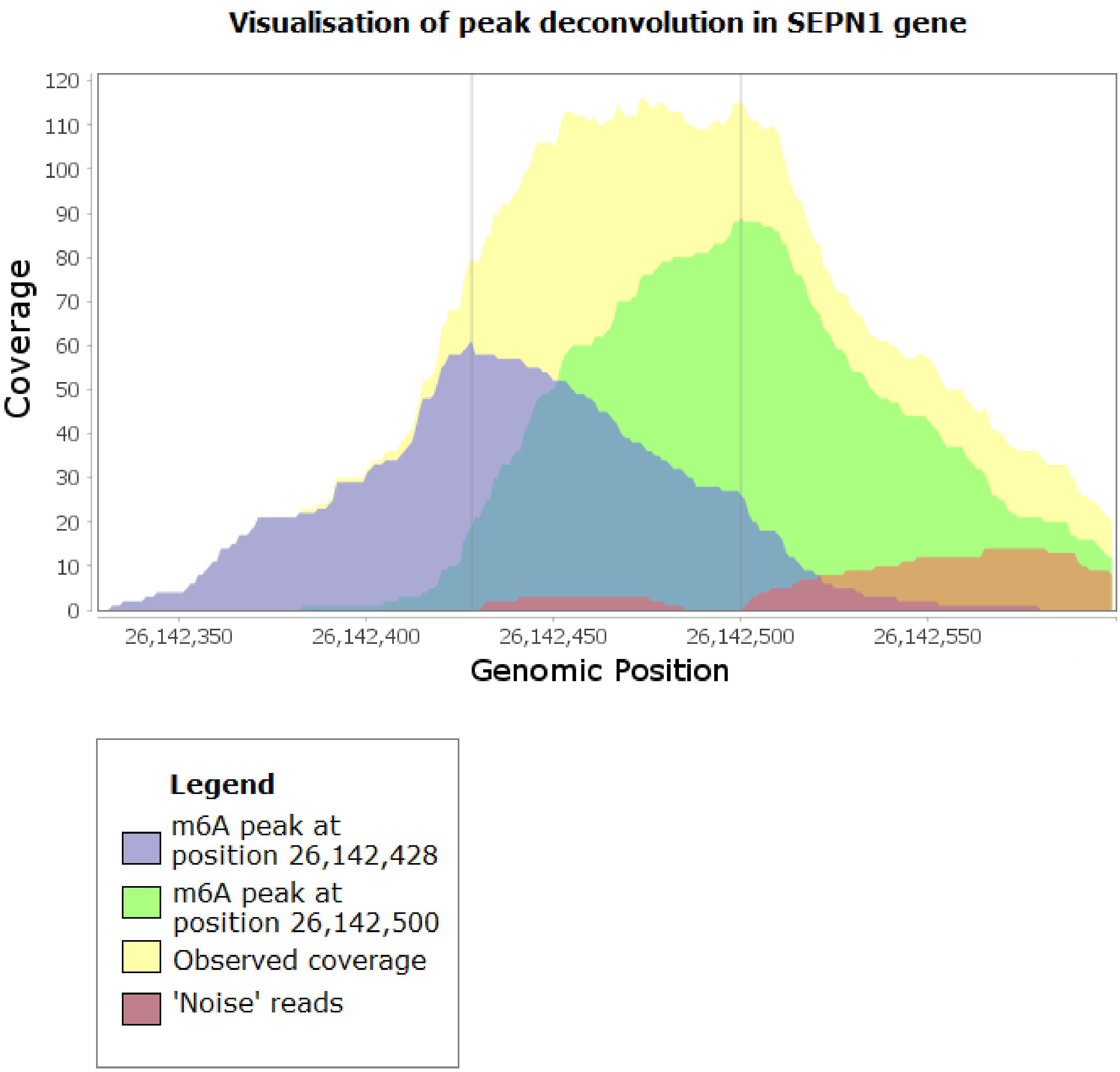

Supplementary Figure 3

Cost/benefit analysis of false-positive m^{6}A filter.

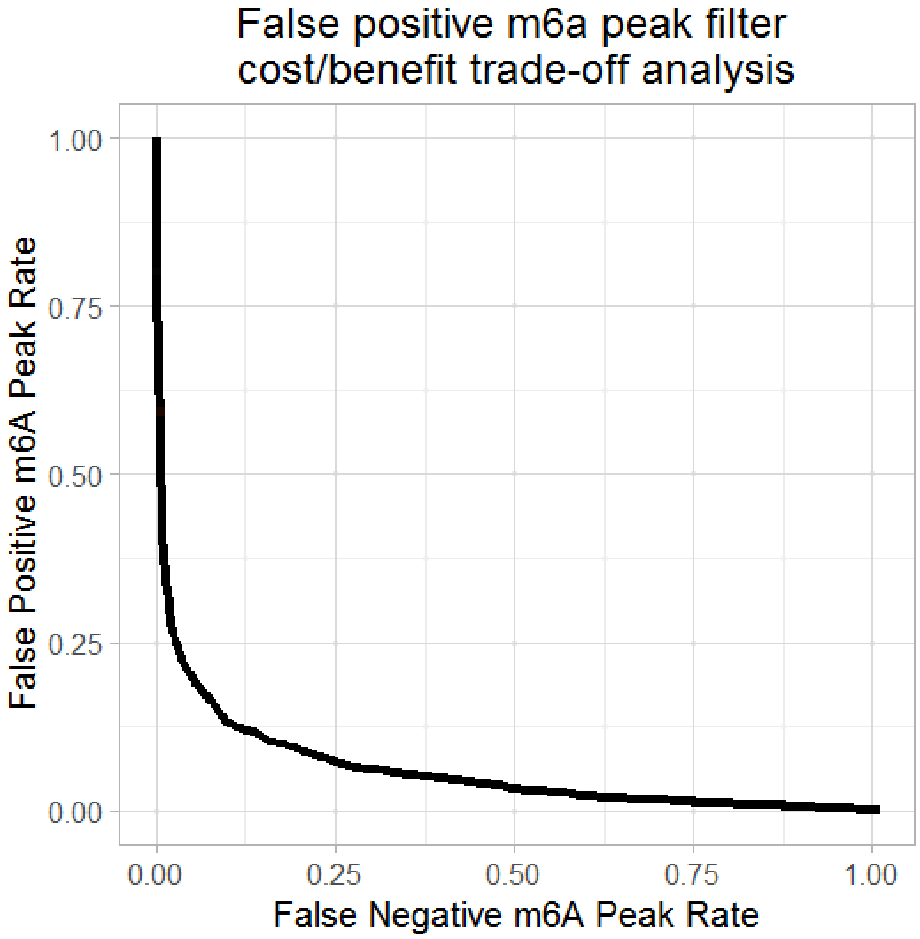

Supplementary Figure 4

Cumulative frequency distribution of detected peak distance to nearest 'RRACH' consensus sequence motif in peaks called by different peak-calling software, assessed on m6A-Seq data from H1299 cells (ArrayExpress Accession: E-GEOD-76367), and HIV infected and control MT4 cells (ArrayExpress Accession: E-GEOD-74016).

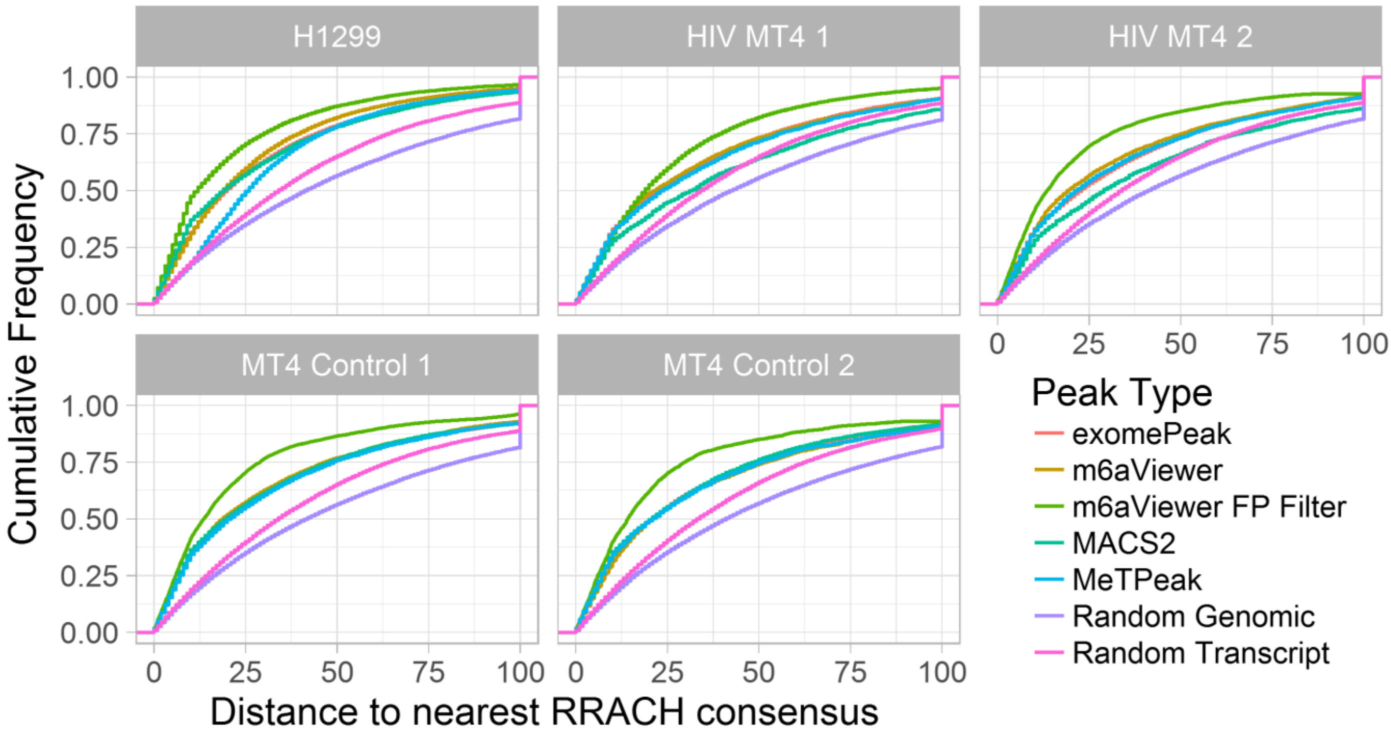

Supplement: Supplemental Material [file supp_058206.116_Supplemental_Figures.pdf]
