# Supplementary material for: m6aViewer: software for the detection, analysis, and visualization of N6-methyladenosine peaks from m6A-seq/ME-RIP sequencing data
Source: RNA. 2017 Oct;23(10):1493–501. doi: 10.1261/rna.058206.116 (PMC5602108; doi:10.1261/rna.058206.116)
Supplement: Supplemental Material [file supp_058206.116_Supplemental_Material.pdf]

Supplementary Methods

1. Peak Deconvolution

1.1 EM approach

Improving peak-calling resolution is important for downstream validation of m^6A sites via PCR as well as additional experiments, such as identification of m^6A reader proteins. Furthermore, it is important to separate individual m^6A sites in experiments which aim to detect methylation changes across different conditions.

When considering the RNA fragment coverage alone, individual RNA fragment positional information is lost, which could be used to inform m^6A residue positions. Consider any region enriched in immunoprecipitated RNA fragment coverage. Reads aligning to such a region are a product of one of several possible cases – antibody binding to one or more m^6A residues; antibody binding to RNA non-specifically; free RNA (or DNA) fragment contamination that can arise from RNA “sticking” to beads or other surfaces; or erroneous read alignment (due to poor read quality or low complexity regions). The latter cases can be considered noise, and with the possible exception of non-specific antibody binding, should constitute a minor fraction of all the reads aligning to an enriched region. Thus, the observed immunoprecipitated fragment coverage distribution can be seen as a mixture of noise and one or more m^6A components. If these components could be deconvoluted, individual as well as multiple m^6A sites in close proximity could be identified more accurately.

The problem can be framed as one of maximum likelihood – what combination of components explains the observed RNA fragment distribution best? Here, expectation maximization (EM) algorithm is used to iteratively compute the likeliest m^6A sites and fit the observed fragment distribution to the model. EM is often used to find maximum likelihood-based approximations of parameters in probabilistic models, and here m^6A positions can be seen as parameters to be estimated in a probabilistic RNA fragment distribution model.

In order to approach the problem of m^6A peak deconvolution in a probabilistic way, a robust way of estimating the probability that a fragment is seen as a result of antibody recognition of a specific site is required. We can assess this in terms of how well each fragment supports the expected coverage distribution around the m^6A site. In the case of an aligned fragment that does not overlap a putative m^6A site, this probability is effectively zero. For all other cases, given only a single aligned fragment as evidence and under (the somewhat inaccurate) assumption of a random fragmentation process, this probability should be uniform. In practice, however, we observe that this is not strictly the case – on investigating well defined, single m6A peaks, we found that there is bias for m6A to occur away from sequenced fragment ends. Thus, this empirical distribution can be used to estimate probabilities in our model. Furthermore, we know that the stochastic process that generates these observations is governed by a non-uniform latent variable –the actual m6A distribution. Thus, the posterior probability that the observed fragment was generated by antibody binding to a putative m6A position can be modelled by including this prior using EM.

For a given region, let $X=(x_0, ..., x_{k-1})$ be a k-sized vector of sequenced RNA fragments aligned to the region, drawn from an unknown mixture of size n of D distributions, each representing either an m^6A site or a noise read cluster. In order to save computational costs, reads aligning to regions with extremely high coverage are down-sampled. We wish to find a set of parameters θ that maximize the log likelihood function:

$$L(\theta) = \ln P(X|\theta)$$

where θ consists of m^6A positions and noise cluster $C \in \{c_0, ..., c_{n-1}\}$ and a corresponding prior probability vector $S \in \{s_0, ..., s_{n-1}\}$ where $\sum_{i=0}^{n} S_i = 1$ and $0 \leq S_i \leq 1$. Here, priors effectively capture differing m^6A stoichiometry at each position - the proportion of methylated RNA molecules at the given position, which influences the observed peak height and therefore mixture proportions. EM approach can iteratively estimate θ while maximising $L(\theta)$ and consists of two steps. During the expectation step, posterior probabilities of each mapped RNA fragment arising from each putative m^6A position in C are computed, given the estimated parameters θ at step t.

$$P(x_i \in C_j \mid \theta_t) = \frac{Sj \cdot P(x_i \mid D, C_j)}{\sum_{j=0}^{n} Sj \cdot P(x_i \mid D, C_j)}$$

The maximisation step then re-estimates parameters θ_t from the posterior probabilities obtained during the expectation step. Each prior probability at step t is estimated as:

$$S_{j,t} = \sum_{i=0}^{k} P(x_i \mid S_{j,t-1}, C_{j,t-1}) \Big/ k$$

and each m^6A position as:

$$C_{j,t} = \frac{\sum_{i=0}^{k} P(x_i \mid S_{j,t-1}, C_{j,t-1}) \cdot C_{j,t-1}}{\sum_{i=0}^{k} P(x_i \mid S_{j,t-1}, C_{j,t-1})}$$

The process iterates for a set number of iterations, or (in most cases) until the algorithm has converged when $L(\theta_t) - L(\theta_{t-1}) < 0.01$, where the likelihood at step t is computed as:

$$L(\theta_t) = \ln\left(\prod_{i=0}^{k} \max_{j=0;j<n} P(x_i|C_j, S_j)\right)$$

where each sequenced read fragment is effectively assigned to either the likeliest m^6A position cluster or a noise cluster. This iterative procedure is visualised in a simulated example below, where observed fragment coverage is shown in red, while peaks fitted at each iteration are shown in black (noise cluster not drawn to aid clarity).

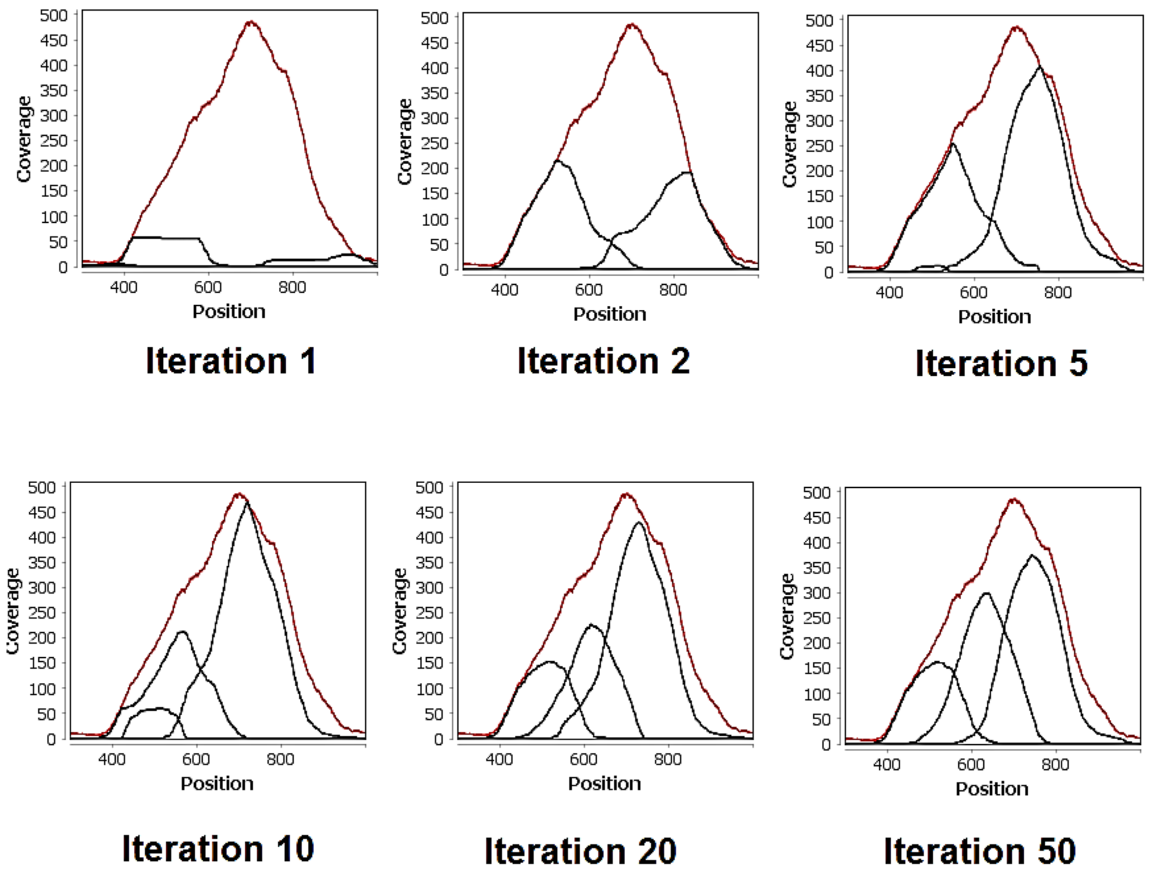

1.2 EM initialization

It is important to initialize EM with good starting values – poor starting values in particular can result in the algorithm becoming 'trapped' at local maxima and therefore failing to converge to the global maximum. EM is frequently initialized randomly, while more robust approaches adopt multiple restart strategies; however, these can be computationally expensive, which is a major concern when trying to apply this approach to whole transcriptome data. Here, a two-step data-guided approach is employed that does not require EM to be run multiple times, while ensuring good initialization values.

As m^6A positions are more likely to occur in regions which have high fragment coverage (i.e. towards the summit of the peak, rather than slopes), initially n positions in a region are chosen based on the RNA fragment coverage values, where n is the number of peaks to be fitted. This selection is done iteratively, such that each read encompassing the previously selected positions is not counted towards the next position; this strategy prevents initialization of multiple positions per peak. The initial selection is then refined based on the reference sequence, as methylation is more likely to occur at a RRACH motif and must occur at an adenosine. All reference positions for 'A', 'AC' and 'RRACH' are extracted, with more weight being given to 'RRACH' and

‘AC’ motifs than just adenosines. Motif weights ensure that a ‘RRACH’ motif, for example, 10 bases away will be prioritized over ‘AC’ 5 away, but if the motif is too far, the nearest ‘AC’ or ‘A’ is used instead. Optimal adjustment of initial coverage-based positions can then be formulated as the assignment problem, where each initial position needs to be matched to the closest motif position in a manner which minimizes the total adjustment distance for all positions. Here, this is solved with the Hungarian algorithm using a distance matrix constructed to represent the bipartite graph between coverage-based positions and sequence motif positions. The prior probability vector is then initialized with read coverage proportions at these positions.

1.3 EM Component Number Estimation

Lastly, the final parameter that needs to be estimated is the number of peaks to be fitted to any given region. Model likelihood increases with the number of components, thus for any given region, the maximum model likelihood could be achieved by fitting an m^6A position for each A in the region, as this would maximize the probabilities for each individual data point. This is clearly a nonsensical result, however. Thus, a method is needed to select an optimal number of components that takes into account model complexity in addition to likelihood. How many clusters to fit in an unsupervised approach is a fundamental problem, which has been widely studied (Fraley and Raftery).

Here, we opt to use Bayesian information criteria (BIC) to adjust model likelihood scores, as it has been previously shown to perform well in the context of EM. Effectively, BIC introduces a penalty for increasing the number of parameters in the model (Schwarz 1978; Hirose et al. 2011):

$$BIC = L(\theta) - 1/2 * k * log\ (n)$$

Where $L(\theta)$ is the log likelihood of the model, k is the number of parameters and n is the size of the data set. Here, for each region BIC is computed for the multiple possible models with increasing component counts and the optimum model is chosen as the one that corresponds to the first BIC maximum. Effectively, this identifies the point where the increase in model likelihood no longer compensates for the increase in model complexity penalty. While Dasgupta and Raftery (Dasgupta and Raftery 1998) suggest estimating a maximum on likely amount of true clusters for the data and computing BIC for all the resulting models, here models are computed iteratively only until a maximum is detected in order to save computation times. For example, in the deconvolution above, BIC is maximized at 3 peaks:

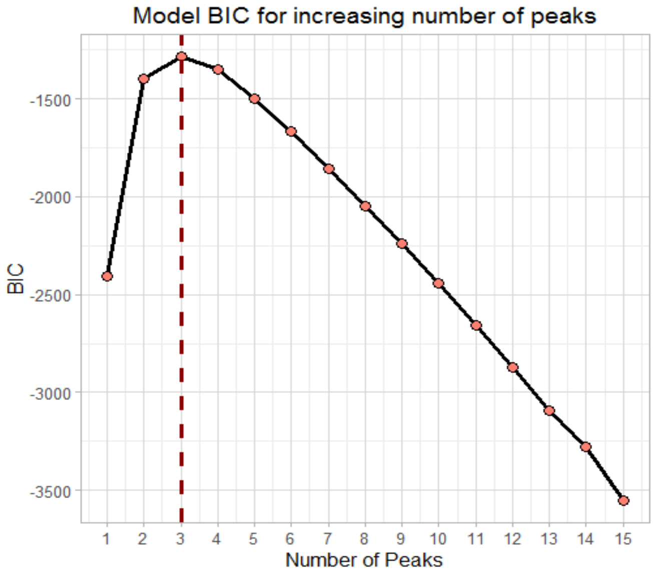

2. False Positive Peak Filter

2.1 Sequence-based MTD model

RNA methyltransferase knockdown and matched control m^{6}A-seq data from HEK293T cells, A549 cells and mouse fibroblast cells were downloaded from ArrayExpress (Kolesnikov et al. 2015), aligned using Star aligner (Dobin et al. 2013) to either hg19 or mm10 reference genomes and sorted and indexed using Samtools (Li et al. 2009). Peak-calling was performed by m6aViewer running in default mode, as the model-based peak-calling involves a sequence-based initialization step which may bias the results. Matched knockdown and control sample sites were intersected and peaks labelled as true positive m^{6}A sites if m^{6}A peak was not detected in the knockdown sample and the gene expression level of the transcript has not decreased so as to prevent detection of the peak. Similarly, a peak was labelled as a technical false positive only if the change in peak enrichment levels between the knockdown and the control was less than 0.5 fold. For the purposes of intersecting the samples, two peaks considered to be the same site in two samples if detected within 50 base pairs of each other. On the other hand, two sites were considered independent sites if they were detected further than 200 base pairs apart in the matched samples. All other sites were considered ambiguous and therefore excluded from the selection in order to obtain the highest quality training set. Using this approach, a training dataset from HEK293T cell line data was obtained, comprising 2098 peaks selected to have a balanced mixture of coding and non-coding sequences. Out of these, 1030 peaks were classed as false positive training instances and 1068 as true positive instances. The datasets obtained from A549 and mouse fibroblast cells was reversed solely for independent testing.

In order to ascertain whether true m^{6}A sites could be differentiated from false positives using only unbiased features that are independent of external data/annotations, an RNA sequence-only model was initially considered. A sequence-only model is attractive in that it can be applied universally in an unbiased manner with only a transcriptome sequence requirement. As such, for each peak in the training dataset, 200 nt RNA sequence surrounding the peak (100 nt each side) was obtained and each such training sequence was represented as a

combination of characters A, G, C, U and M, where M represents the putative methylated adenosine position in the sequence.

Markov chains are ideally suited for representing RNA sequence data; however higher order Markov models can be computationally intensive and require increasingly large training datasets with increasing order to combat the uncertainty of estimating the transition probability matrix values for most large *k*mers. Mixture Transition Distribution (Berchtold and Raftery 2002) (MTD) models has been utilized in sequence modelling of DNA methylation sites (Seifert et al. 2012) and provide an alternative way of estimating the transition probabilities for high order Markov chains. Rather than computing the probability of observing a particular base after a specific kmer, which cannot be estimated accurately when the kmer is large, the probability is estimated as a combination of different 'lag' probabilities. This is a computationally attractive model, as the transition probability matrix required grows linearly with increasing Markov chain order, rather than exponentially.

We further split our training dataset into coding and non-coding sequences in order to avoid any potential biases, as m^6A can be enriched in UTRs (thus avoiding unintentionally building a classifier that specifically identifies UTR sequences, rather than m^6A containing sequences!). Several MTD models were constructed and their predictive power assessed using 10-fold cross validation. The figure below shows the AUC (Area under ROC curve) achieved by sequence models of increasing order:

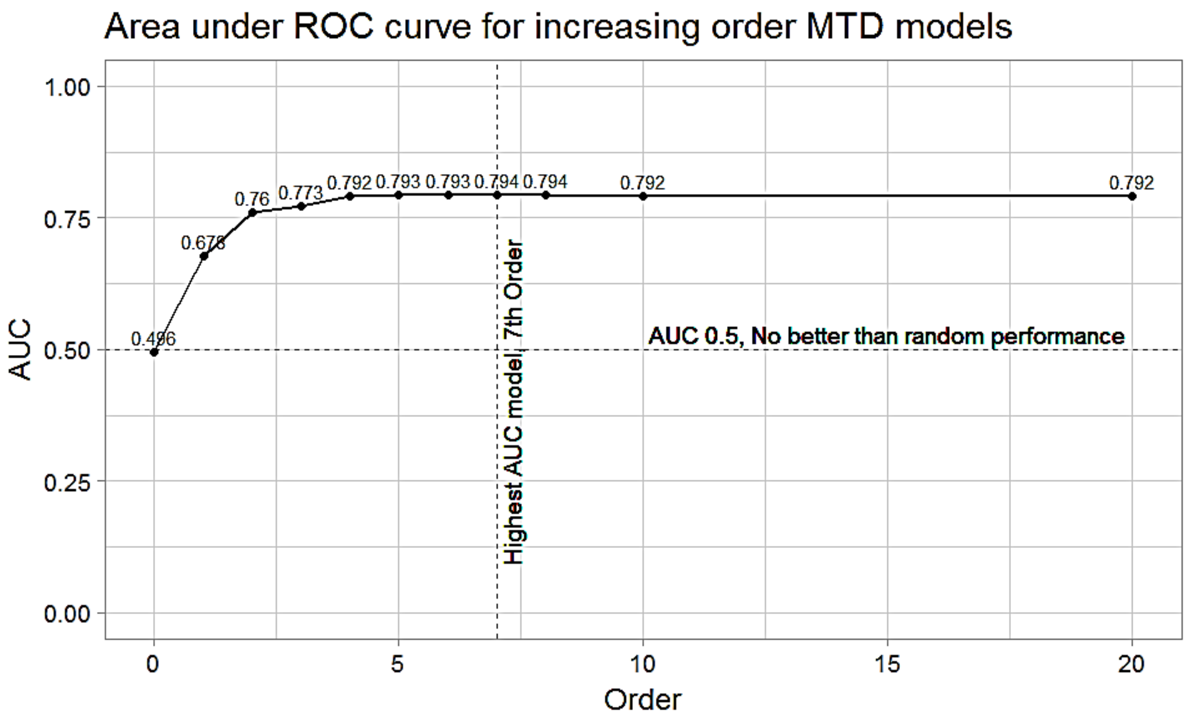

The best performance was achieved by 7th Order MTD model, with AUC of 0.794:

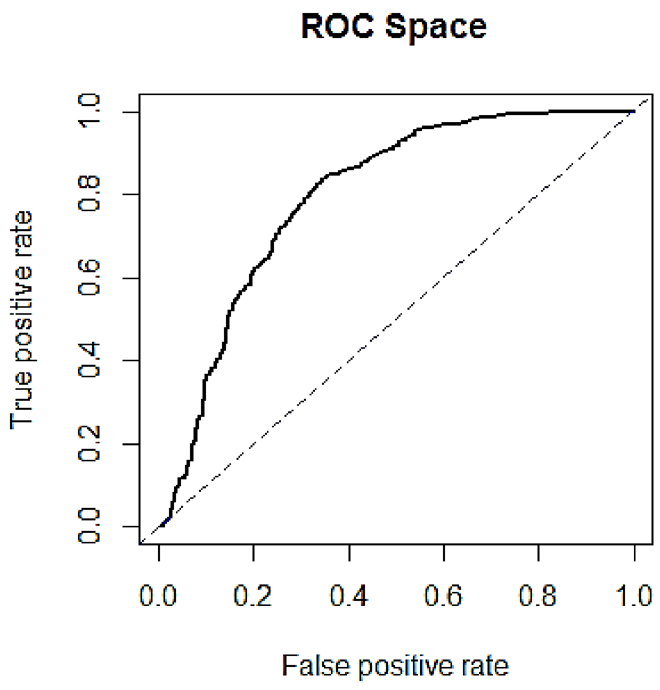

AUC of 0.794 indicates that the MTD sequence model can differentiate between technical false positive peak sequences and m^6A peak sequences in a high proportion of all cases. However, the cost-benefit trade-off is not acceptable for practical applications. Thus, to be applicable for real data m6a-seq data, the sequence classifier required further improvement.

We next considered incorporating secondary sequence structure predictions from RNAfold package (Lorenz et al. 2011) into our MTD model by increasing the MTD model dictionary size and representing each base as either single stranded or double stranded. We found that this did not improve the classification power of our best model:

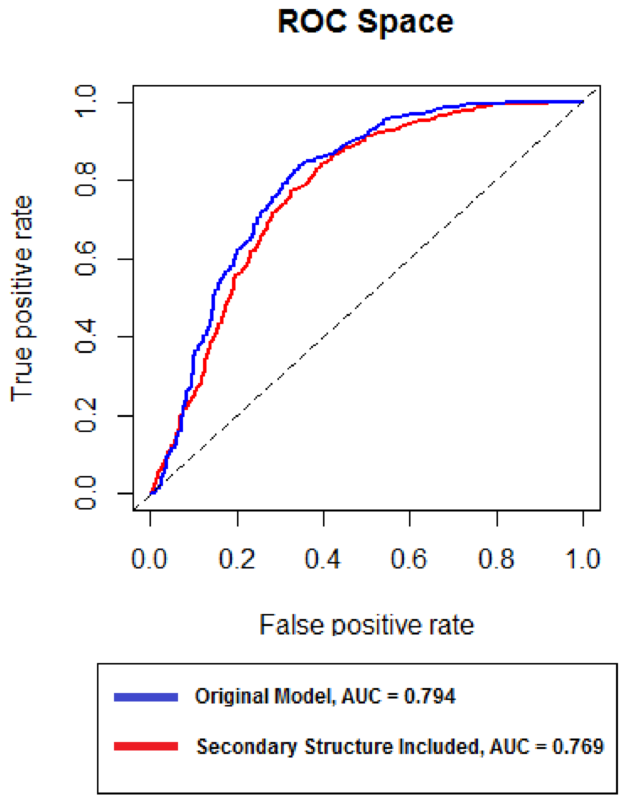

2.2 Random Forest Feature-based model

As the sequence-based model could not be further improved by incorporating secondary structure based information, a feature-based model was considered next. There are a number of features in additional to sequence information and RNA secondary structure which could be predictive of m^6A status, including transcript information, sequence composition, sequencing data features surrounding the peak or conservation information. These were obtained for our training and testing datasets and investigated (see **Supplementary Data** for detailed list). We trained a random forest classifier using a subset of features selected from a greedy stepwise feature search, where at each step a smaller a model was trained using a subset of all features and evaluated using 10-fold cross validation. At each step, another most informative feature was added, until the addition of extra features resulted in the decrease in the overall performance. Consistently with the MTD sequence model, the expanded RNA secondary structure features were not selected as informative for the final model, suggesting that either the error rate in RNA secondary structure prediction is too high, RNA secondary structure is not important for RNA adenosine methylation, or a similar RNA secondary structure is present at non-specific antibody binding sites to that of actual m^6A sites, which could also be an antibody site recognition factor.

3.0 Sequence Data Processing

m6aViewer subdivides the reference genome and processes the sequence data in smaller blocks. This approach enables both effective memory management for m^6A-seq data of all transcriptome sizes, as well as parallel processing of blocks on modern, multi-core hardware. Performance gains from multi-threading and increasing available memory are summarised below:

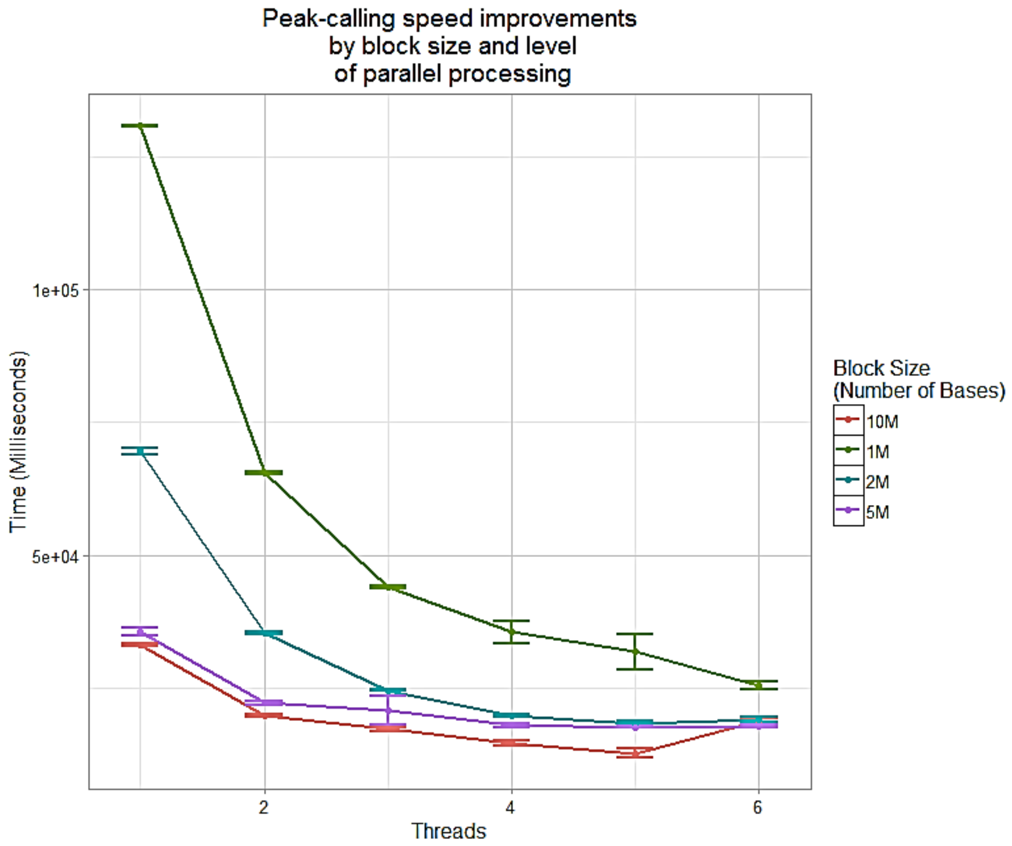

Increasing both block size and parallelization can greatly increase peak-calling speed. Similar gains in speed can be achieved by initially doubling the memory use (block size) or the number of parallel processing threads (1Thread, 1M Block: mean= 130772 Ms, 2 Threads, 1M Block: mean=65427 Ms, 1 Thread, 2M Block: mean= 69560.6 Ms). Both increasing memory use (block size) and the number of processing threads have diminishing returns. All tests were repeated 10 times using a Centos machine (16 cores @ 2.27 GHz, 64GB RAM, ST3500630NS HDD) using paired-end in-house m^6A-Seq data (chromosome 1 alignments only, 11.46 million IP reads and 6.96 million INPUT reads).

For each block, slightly overlapping block ends are used to avoid peak-calling errors at block boundaries and sequence data is read in from BAM file in parallel by each worker thread. Each read (or read pair) is subject (by default, can be turned off) to quality filtering to remove optical/PCR duplicates, secondary alignments, low quality alignments and improper read pairs. For the filtering step to work, input BAM files require appropriate flags to be set, for example by running software such as Picard tools to mark duplicate reads.

4.0 Peak-calling

4.1 Signal Smoothing

Fragment coverage is obtained from all fragments passing quality filtering. The resulting signal is smoothed using a sliding window approach in order to remove small, local irregularities and facilitate the detection of

peaks using a local maxima approach. For each reference base position, $C_{x,}$ data smoothing is performed as a local mean using *n* (here, *n*= 20) data points surrounding it:

$$C_x = \frac{1}{n} \sum_{i=-(n-1)/2}^{(n-1)/2} C_{x-i}$$

4.2 Detection of Local Maxima

Local maxima(peaks) and local minima (valleys between overlapping peaks) are subsequently identified from smoothed coverage by searching the coverage array for gradient inversion events. A potential peak is thus initially defined as a position *i* in the smoothed coverage array C, where $C_i - {}_{Ci-1,\ldots,\ i-n} > 0$ && $C_i - C_{i+1,\ldots,\ i+m} > 0$, where *n* and *m* are either the last prior (or first subsequent) gradient change event position detected (if greater than 1/10th of expected peak width, to account for overlapping peaks but also prevent detection of small irregularities in coverage distribution), half the expected peak width or last (or next) position with 0 read coverage, whichever occurs closest. For cases where peak summits are flat – i.e. local maxima spans multiple bases - the putative peak position is defined as the central point.

4.3 p-values and FDR calculations

Each putative peak identified is tested against the null hypothesis that the read distribution in the immunoprecipitated sample is not higher than that in the control. The total number of fragments for the peak region in IP and INPUT samples are counted as the number of fragments aligning to (but not necessarily wholly contained within) the peak region. The peak region is defined as the region encompassing the number of bases equal to the sequenced fragment length to each side of the detected maximum; in cases of peak overlap, the region boundary to the overlapping side(s) of the peak is defined as a mid-point between the two peaks. Respective contingency tables are thus computed from the total IP and INPUT fragments at the putative peak position and the total IP and INPUT library size. Alternatively, local background can be used in this calculation instead, using fragment counts at peak position and total reads aligning to the respective transcript.

Computed p-values can be subjected to Benjamini-Hochberg (Benjamini and Hochberg 1995) or Bonferroni (Armstrong 2014) multiple testing corrections, with peaks below a user-defined significance level retained (default: 0.05). Alternatively, FDR can be estimated and controlled by treating the INPUT sample as IP and performing peak detection in order to obtain an empirical p-value distribution from the switched IP and INPUT samples. The FDR of peak p-values can then be estimated from the obtained distribution and represents the chance of seeing an equivalent read enrichment in the RNA-Seq control data. While this is not an ideal measure, in practice it provides a cut-off that is less stringent than Bonferroni correction, but more stringent than Benjamini-Hotchberg.

References

Armstrong RA. 2014. When to use the Bonferroni correction. *Ophthalmic Physiol Opt* **34**: 502–8. http://www.ncbi.nlm.nih.gov/pubmed/24697967 (Accessed May 5, 2015).

Benjamini Y, Hochberg Y. 1995. Controlling The False Discovery Rate - A Practical And Powerful Approach To Multiple Testing. *J R Stat Soc Ser B Methodol* **57**: 289–300. http://www.researchgate.net/publication/221995234_Controlling_The_False_Discovery_Rate_-_A_Practical_And_Powerful_Approach_To_Multiple_Testing (Accessed October 1, 2015).

Berchtold A, Raftery A. 2002. The Mixture Transition Distribution Model for High-Order Markov Chains and Non-Gaussian Time Series. *Stat Sci* **17**: 328–356. http://projecteuclid.org/euclid.ss/1042727943 (Accessed March 9, 2016).

Dasgupta A, Raftery A. 1998. Detecting features in spatial point processes with clutter via model-based clustering. *J Am Stat*. http://amstat.tandfonline.com/doi/abs/10.1080/01621459.1998.10474110 (Accessed August 3, 2016).

Dobin A, Davis CA, Schlesinger F, Drenkow J, Zaleski C, Jha S, Batut P, Chaisson M, Gingeras TR. 2013. STAR: ultrafast universal RNA-seq aligner. *Bioinformatics* **29**: 15–21. http://bioinformatics.oxfordjournals.org/content/29/1/15 (Accessed July 13, 2014).

Fraley C, Raftery AE. How Many Clusters? Which Clustering Method? Answers Via Model-Based Cluster Analysis.

Hirose K, Kawano S, Konishi S, Ichikawa M. 2011. Bayesian information criterion and selection of the number of factors in factor analysis models. *J Data Sci*. http://www.jdsruc.org/upload/JDS-682(2011-04-01164527).pdf (Accessed April 13, 2016).

Kolesnikov N, Hastings E, Keays M, Melnichuk O, Tang YA, Williams E, Dylag M, Kurbatova N, Brandizi M, Burdett T, et al. 2015. ArrayExpress update--simplifying data submissions. *Nucleic Acids Res* **43**: D1113-6. http://europepmc.org/articles/PMC4383899 (Accessed April 21, 2015).

Li H, Handsaker B, Wysoker A, Fennell T, Ruan J, Homer N, Marth G, Abecasis G, Durbin R. 2009. The Sequence Alignment/Map format and SAMtools. *Bioinformatics* **25**: 2078–9. http://www.pubmedcentral.nih.gov/articlerender.fcgi?artid=2723002&tool=pmcentrez&rendertype=abstract (Accessed July 9, 2014).

Lorenz R, Bernhart SH, Höner Zu Siederdissen C, Tafer H, Flamm C, Stadler PF, Hofacker IL. 2011. ViennaRNA Package 2.0. *Algorithms Mol Biol* **6**: 26. http://www.pubmedcentral.nih.gov/articlerender.fcgi?artid=3319429&tool=pmcentrez&rendertype=abstract (Accessed May 20, 2016).

Schwarz G. 1978. Estimating the Dimension of a Model. *Ann Stat* **6**: 461–464. http://projecteuclid.org/euclid.aos/1176344136 (Accessed August 3, 2016).

Seifert M, Cortijo S, Colomé-Tatché M, Johannes F, Roudier F, Colot V. 2012. MeDIP-HMM: genome-wide

identification of distinct DNA methylation states from high-density tiling arrays. *Bioinformatics* **28**: 2930–9. http://www.ncbi.nlm.nih.gov/pubmed/22989518 (Accessed August 4, 2016).
